# Diagnostic accuracy and cut-off values of serum leucine-rich alpha-2 glycoprotein for Crohn’s disease activity in the small bowel

**DOI:** 10.1007/s00535-025-02223-1

**Published:** 2025-02-14

**Authors:** Muneyori Okita, Kento Takenaka, Fumihito Hirai, Shinya Ashizuka, Hideki Iijima, Shigeki Bamba, Toshimitsu Fujii, Kenji Watanabe, Yosuke Shimodaira, Hisashi Shiga, Sakiko Hiraoka, Toshihiro Inokuchi, Takeshi Yamamura, Ryo Emoto, Shigeyuki Matsui

**Affiliations:** 1 Department of Biostatistics, Nagoya University Graduate School of Medicine, 65 Tsurumai, Showa-ku, Nagoya, Aichi 466-8550 Japan; 2 Department of Gastroenterology and Hepatology, Institute of Science Tokyo, Tokyo, Japan; 3 Department of Gastroenterology and Medicine, Fukuoka University Faculty of Medicine, Fukuoka, Japan; 4 Osaka International Medical & Science Center, Osaka Keisatsu Hospital, Osaka, Japan; 5 Department of Fundamental Nursing, Shiga University of Medical Science, Shiga, Japan; 6 Department of Internal Medicine for Inflammatory Bowel Disease, Toyama University, Toyama, Japan; 7 Department of Gastroenterology and Neurology, Akita University Graduate School of Medicine, Akita, Japan; 8 Division of Gastroenterology, Tohoku University Graduate School of Medicine, Miyagi, Japan; 9 Department of Gastroenterology and Hepatology, Dentistry and Pharmaceutical Sciences, Okayama University Graduate School of Medicine, Okayama, Japan; 10 Research Center for Intestinal Health Science, Okayama University, Okayama, Japan; 11 Department of Gastroenterology and Hepatology, Nagoya University Graduate School of Medicine, 65 Tsurumai, Showa-ku, Nagoya, Aichi 466-8550 Japan

**Keywords:** LRG, Biomarker, Crohn’s disease

## Abstract

**Background:**

Small bowel (SB) lesions in Crohn’s disease (CD) are often asymptomatic despite being highly active. Fecal calprotectin (FC) is the most widely used biomarker of CD activity, but its drawbacks include a large intra-individual sample variability and the burden of collecting stool samples. Meanwhile, serum leucine-rich alpha-2 glycoprotein (LRG) has recently attracted attention as a biomarker that can address the limitations of FC. This study determined the diagnostic accuracy of LRG and its cut-off values for diagnosing CD activity in SB.

**Methods:**

This was a retrospective, multi-center study of CD patients undergoing retrograde balloon-assisted endoscopy. For ileal- and ileocolonic-type patients with a colon SES-CD score of 0, we estimated the receiver operating characteristic curve of LRG and determined the cut-off value to achieve a target sensitivity level of 80%.

**Results:**

Among 285 patients with SB lesions, LRG had an area under the curve (AUC) of 0.72 (95% CI 0.67–0.78) with a sensitivity of 80.2% and specificity of 47.2% for a cut-off value of 10.5 when diagnosing endoscopic remission (modified SES-CD ≤ 3), while it had an AUC of 0.72 (95% CI 0.65–0.78) with a sensitivity of 81.2% and specificity of 46.2% for a cut-off value of 10.1 when diagnosing complete ulcer healing (modified SES-CD ≤ 1).

**Conclusion:**

LRG was effective for diagnosing CD activity in SB, specifically with cut-off values of 10.5 and 10.1 for endoscopic remission and complete ulcer healing, respectively. A future prospective validation study will assess its clinical utility.

**Graphical abstract:**

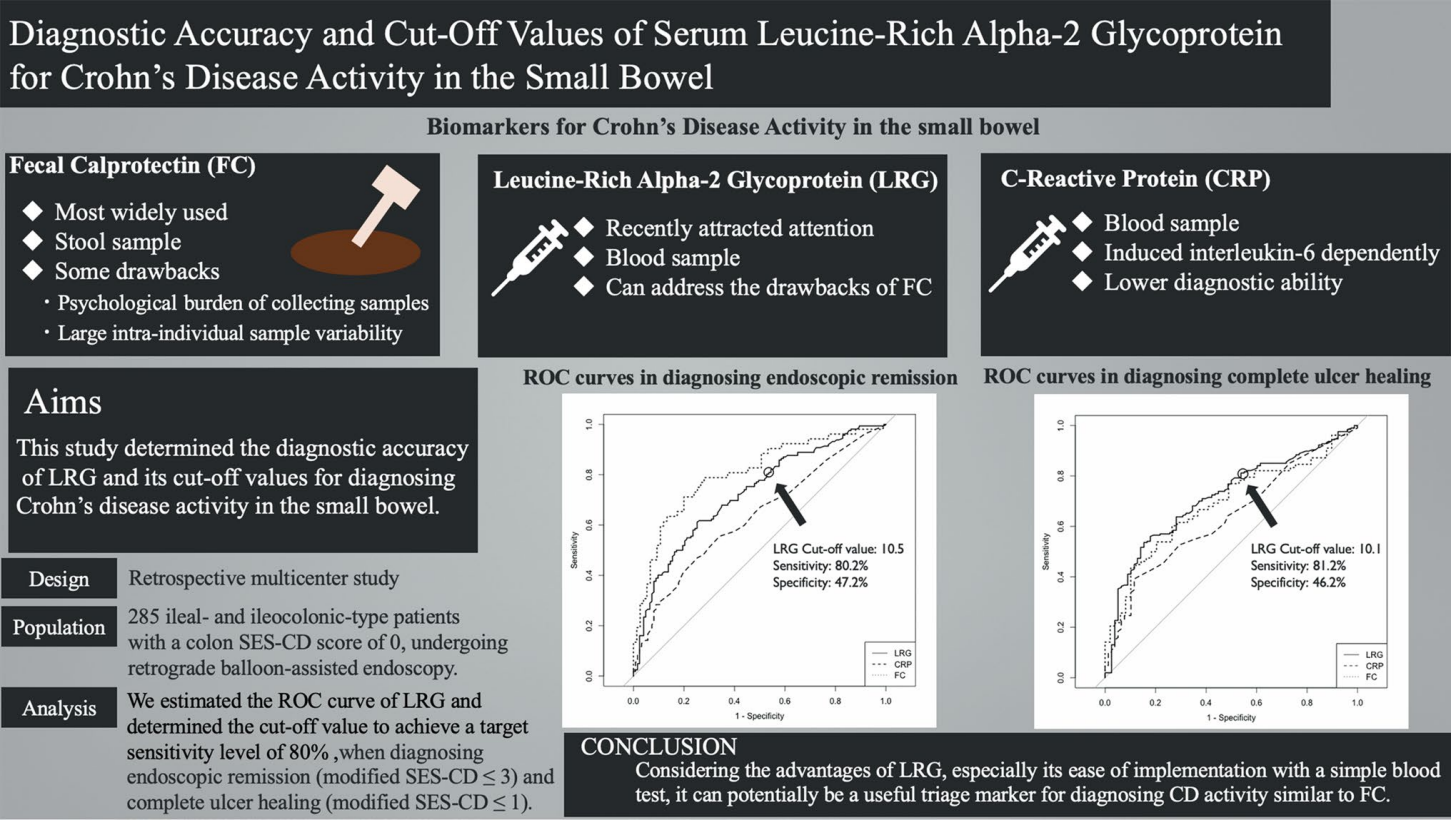

**Supplementary Information:**

The online version contains supplementary material available at 10.1007/s00535-025-02223-1.

## Introduction

Colorectal lesions in Crohn’s disease (CD) are known to cause diarrhea, abdominal pain, and other symptoms that easily interfere with daily life. However, small bowel (SB) lesions in CD are often asymptomatic despite being highly active, and thus the lesions often progress unnoticed, resulting in the formation of stenosis or fistula that requires surgery [1, 2]. Accurately assessing the disease activity of SB lesions is necessary to improve the prognosis of patients with CD. Balloon-assisted endoscopy (BAE) is one of the best tools for accurately evaluating luminal lesions of the SB, but it is invasive and financially burdensome [3]. Therefore, there is a need for an accurate triage marker which can determine the need for further evaluation with BAE [4].

Currently, fecal calprotectin (FC) is the most widely used triage marker for CD activity worldwide [5, 6]. However, FC has several drawbacks, including a large intra-individual sample variability (i.e., within-sample variability, diurnal variability, and day-to-day variability), large age-related variation, and psychological burden on the patient related to submitting stool samples; these can result in low compliance and delayed therapeutic intervention [7–9]. Conversely, serum leucine-rich alpha-2 glycoprotein (LRG) [10] has recently attracted attention, especially in Japan. Being a blood biomarker LRG can avoid some of the drawbacks of FC mentioned earlier. In 2023, a systematic review and meta-analysis [11] reported the diagnostic accuracy of LRG for CD activity, with a sensitivity of 77.0% (95% CI 67.8–84.2) and specificity of 81.1% (95% CI 72.6–87.4%). However, this meta-analysis did not focus exclusively on SB lesions in CD.

This retrospective study evaluated the accuracy of LRG and its cut-off values in diagnosing CD activity in the SB. This project is affiliated with a nation-wide research group, the Health and Labor Sciences Research Grants for research on intractable diseases from the Ministry of Health, Labor and Welfare of Japan.

## Methods

### Patients

This was a retrospective, multi-center study of CD patients. The inclusion criteria were as follows: (1) diagnosed with CD based on clinical, endoscopic, and histological criteria; (2) with a history of outpatient visits or hospitalization at our hospital and collaborating institutions; (3) age≧18 years; (4) had LRG values measured by Nanopia (Sekisui Medical, Tokyo, Japan); and (5) the ileum can be adequately evaluated via retrograde BAE (EN-580XP, FUJIFILM Corporation, Japan; SIF-Q260, OLYMPUS CORPORATION, Japan) or long-narrow colonoscopy (PCF-H290, OLYMPUS CORPORATION, Japan) including selective contrast examination at the deepest point reachable by the endoscope. Conversely, the exclusion criteria were as follows: (1) having an interval of > 30 days between endoscopy and LRG measurement; (2) treatment modification between endoscopy and LRG measurement; (3) contraindicated for BAE because of severe SB strictures detected radiographically, (4) severe extraintestinal manifestations and anal lesions; and (5) any cancer. No exclusion criteria were established for disease duration, and initial diagnostic CD patients were also included in this study. Notably, it was expected that a limited number of patients would have an interval of less than 30 days between endoscopy and LRG measurement, because of the insurance-related time constraints between the two examinations. At all institutions, CD was diagnosed based on the diagnostic criteria of the Ministry of Health, Labor, and Welfare (i.e., “Research on Intractable Inflammatory Bowel Disorders”). This study protocol was approved by the Ethics Committee at Nagoya University Hospital (2023–0449), and the study was conducted in accordance with the Declaration of Helsinki. This study was conducted retrospectively and we guaranteed participants an opportunity for rejection through information disclosure.

### Characteristics and outcome variables

The following demographic data were collected: age, sex, disease duration, history of major intestinal surgery (i.e., intestinal resection or strictureplasty), smoking (i.e., current smoker, previous smoker, or non-smoker), anal disease (i.e., perianal abscess or anal fistula), liver cirrhosis, and complications of other immunological diseases. In addition, medication data for the following drugs were collected: non-steroidal anti-inflammatory drugs, steroids, antiplatelet drugs, immunomodulators, infliximab, adalimumab, vedolizumab, ustekinumab, risankizumab, and upadacitinib. All medication data were related to current medication use; the history of previous medication use was unaccounted for. In addition, various liver diseases may affect LRG values (since the liver produces LRG), but the data regarding this were scarce. Thus, only data on liver cirrhosis were collected.

The following outcome variables were collected: Crohn’s Disease Activity Index (CDAI) score, Patient-Reported Outcome (PRO-2), modified Simple Endoscopic Score for CD (modified SES-CD) [12], presence of fistula, location** (**L1: ileal, L2: colonic, and L3: ileocolonic), behavior** (**B1: inflammatory**,** B2: stricturing, and B3: penetrating), as well as the levels of LRG, C-reactive protein (CRP), FC, albumin (Alb), aspartate aminotransferase (AST), alanine aminotransferase (ALT), and hemoglobin (Hb). For these outcome variables, if data were obtained from the same patient multiple times, only the first dataset was used for analysis. Biomarker data were measured on the same day as LRG. If such data did not exist, biomarker data were measured on the date closest to the date of endoscopy. The standard body weight for CDAI calculation was determined as follows: (height in meters)^2^ × 22. Only Bristol stool scale 6 or 7 was counted for defecation frequency when calculating CDAI and PRO-2 scores. PRO-2 scores were calculated by simple addition of CDAI-1 and CDAI-2 scores without weighting.

### Imaging modalities

This study included patients who underwent retrograde BAE, or whose ileum could be adequately evaluated via long-narrow colonoscopy including selective contrast examination from the deepest point of endoscopic reach. Endoscopic evaluations were performed using the modified SES-CD. The SB was divided into following three segments: jejunum, proximal ileum, and terminal ileum, defined as the portion of the ileum observable by colonoscopy. In addition, considering that the length of the SB is ≤ 600 cm, and that the jejunum and ileum are roughly the same length, the terminal ileum, proximal ileum, and jejunum were defined as the segments ≤ 10 cm, 10–300 cm, and 300–600 cm from the ileocecal valve, respectively [12]. The right colon was defined as the segment including the cecum with the ileocecal valve and the ascending colon until the hepatic flexure. The transverse colon was defined as the segment between the hepatic and splenic flexures. The left colon was defined as the segment including the descending colon and sigmoid colon until the rectosigmoid junction; its continuation until the anal side was defined as the rectum.

For the most severe lesion in each segment, a score of 0–3 was given for the following variables: ulcer size, proportion of ulcerated surface, and proportion of affected surface. Stenosis was not included in the modified SES-CD score because this study focused on the relationship between small bowel mucosal lesion activity and LRG levels. The total score obtained for each segment was summed to obtain the modified SES-CD. Modified SES-CD scores of ≤ 3 and ≤ 1 indicated endoscopic remission and complete ulcer healing, respectively [13]. Many of the institutions participating in this study do not routinely calculate the modified SES-CD score. Therefore, one gastroenterologist at each facility who is an expert of BAE reviewed the endoscopic findings from BAE and calculated the modified SES-CD score, ensuring that patient background and laboratory data were blinded to the person performing the calculation.

### Combination of target population and endoscopic activity

To assess the diagnostic ability of LRG for SB lesions, the target population of SB patients were set as ileal-type and ileocolonic-type patients, both with a colon SES-CD score of 0. In addition, we also set another target population which includes both SB and colon patients, seeing that it would be worthwhile to report the diagnostic ability of LRG in both types of lesions. For all patients, the diagnostic ability of LRG was evaluated based on endoscopic remission and complete ulcer healing.

### Statistical analysis

For all combinations of the two target populations (i.e., SB patients alone, SB and colon patients) and two outcome variables of endoscopic activity (i.e., endoscopic remission, complete ulcer healing), the following analyses were conducted. A receiver operating characteristic (ROC) curve of LRG was estimated to evaluate its diagnostic accuracy for CD activity. Since no obvious factor is strongly correlated with CD activity (as confirmed on multivariate logistic regression analysis), the primary analysis involved estimating the ROC curve of LRG without stratification by any factor.

In determining the cut-off values of LRG, we placed an emphasis on sensitivity, because having a high sensitivity minimizes false negatives. This means that a negative LRG result would suggest a high probability of CD inactivity, and thus BAE can be avoided. Conversely, to address the increased false positives, when the LRG is positive, other clinical findings will be considered to determine if BAE should be performed. We targeted a sensitivity of 80% based on previous studies on FC, reporting sensitivities of 82.4% [5] and 75.0% [6]. The ROC curves of CRP and FC were also estimated, and the differences in the area under curve (AUC) between LRG and CRP or FC were evaluated via two-sided Delong’s test. All analyses were performed using R version 4.2.1.

## Results

### Patient characteristics and endoscopic findings

This study included 285 patients with CD affecting the SB, who were recruited from 6 centers, including 5 university hospitals (Akita University Hospital, Akita; Tohoku University Hospital, Miyagi; Tokyo Medical and Dental University Hospital, Tokyo; Nagoya University Hospital, Aichi; Okayama University Hospital; Okayama) and 1 community hospital (Osaka Keisatsu Hospital, Osaka); their baseline characteristics are summarized in Table 1. There were 154 (54.0%) and 131 (46.0%) patients with ileal-type and ileocolonic-type CD, respectively. Inflammatory behavior, stricturing, and penetrating behavior were observed in 64 (22.5%), 175 (61.4%), and 46 (16.1%) patients, respectively. The median (interquartile range [IQR]) LRG, CRP, and FC values were 12.6 (9.7–17.4) μg/mL, 0.1 (0.0–0.2) mg/dL, and 180.4 (57.7–453.5) μg/g, respectively. Endoscopic remission (modified SES-CD ≤ 3) and complete ulcer healing (modified SES-CD ≤ 1) were achieved in 123 (43.2%) and 78 (27.4%) patients, respectively. The total modified SES-CD score had a median (IQR) of 4 (1–8). The boxplots of modified SES-CD for SB patients are shown in Supplementary Fig. 1.

**Table 1:** Baseline characteristics and endoscopic findings

Variables	SB patients (*n* = 285)	SB and colon patients (*n* = 478)
Age (years)	43 (34–53)	40 (30–50)
Male sex	209 (73.3%)	344 (72.0%)
Disease duration (years)	11 (5–20)	10 (4–19)
Current smokers	26 (9.1%)	45 (9.4%)
History of major intestinal surgery	154 (54.0%)	233 (48.7%)
Perianal disease (perianal abscess, anal fistula)		
Active	10 (3.5%)	35 (7.3%)
Inactive	71 (24.9%)	146 (30.5%)
No history	204 (71.6%)	297 (62.2%)
Complication of liver cirrhosis	1 (0.4%)	1 (0.2%)
Complications of other immunological diseases	3 (1.1%)	6 (1.3%)
Location		
L1: Ileal	154 (54.0%)	182 (38.1%)
L2: Colonic	0 (0.0%)	28 (5.9%)
L3: Ileocolonic	131 (46.0%)	268 (56.0%)
Behavior		
B1: Inflammatory	64 (22.5%)	140 (29.3%)
B2: Stricturing	175 (61.4%)	250 (52.3%)
B3: Penetrating	46 (16.1%)	88 (18.4%)
Crohn’s disease activity index (CDAI)	63 (22 to 138)	68 (25 to 137)
PRO-2 score	7 (0 to 21)	7 (0 to 21)
Medication		
Non-steroidal anti-inflammatory drugs	4 (1.4%)	7 (1.5%)
Steroids	23 (8.1%)	38 (7.9%)
Antiplatelet drugs	0 (0.0%)	1 (0.2%)
Immunomodulators	118 (41.4%)	185 (38.7%)
Infliximab	70 (24.6%)	99 (20.7%)
Adalimumab	65 (22.8%)	130 (27.2%)
Vedolizumab	13 (4.6%)	25 (5.2%)
Ustekinumab	50 (17.5%)	82 (17.2%)
Risankizumab	1 (0.4%)	4 (0.8%)
Upadacitinib	1 (0.4%)	1 (0.2%)
Laboratory data		
Leucine-rich alpha-2 glycoprotein (μg/mL)	12.6 (9.7–17.4)	13.9 (10.5–19.2)
C-reactive protein (mg/dL)	0.1 (0.0–0.2)	0.1 (0.0–0.3)
Fecal calprotectin (μg/g)	180.4 (57.7–453.5)	270.7 (79.6–786.0)
Albumin (g/dL)	4.1 (3.8–4.4)	4.1 (3.8–4.4)
Hemoglobin (g/dL)	13.9 (12.4–14.9)	13.8 (12.3–14.8)
BAE findings		
Modified SES-CD score ≤ 1	78 (27.4%)	100 (20.9%)
Modified SES-CD score ≤ 3	123 (43.2%)	164 (34.3%)
Total modified SES-CD score	4 (1–8)	6 (2–10)

^*^Continuous variables were expressed as median (interquartile range: IQR) and binary variables as absolute sample size (percentage)

The baseline characteristics and endoscopic findings of the 478 SB and colon patients, including 285 SB-only patients, are also summarized in Table 1. There were 182 (38.1%), 28 (5.9%), and 268 (56.0%) patients with ileal-type, colonic-type, and ileocolonic-type CD, respectively. Inflammatory behavior, stricturing, and penetrating behavior were observed in 140 (29.3%), 250 (52.3%), and 88 (18.4%) patients, respectively. The median (interquartile range [IQR)] LRG, CRP, and FC values were 13.9 (10.5–19.2) μg/mL, 0.1 (0.0–0.3) mg/dL, and 270.7 (79.6–786.0) μg/g, respectively. Endoscopic remission (modified SES-CD ≤ 3) and complete ulcer healing (modified SES-CD ≤ 1) were achieved in 164 (34.3%) and 100 (20.9%) patients, respectively. The total modified SES-CD score had a median (IQR) of 6 (2–10). The boxplots of modified SES-CD for SB and colon patients are shown in Supplementary Fig. 2.

**Fig. 1:**
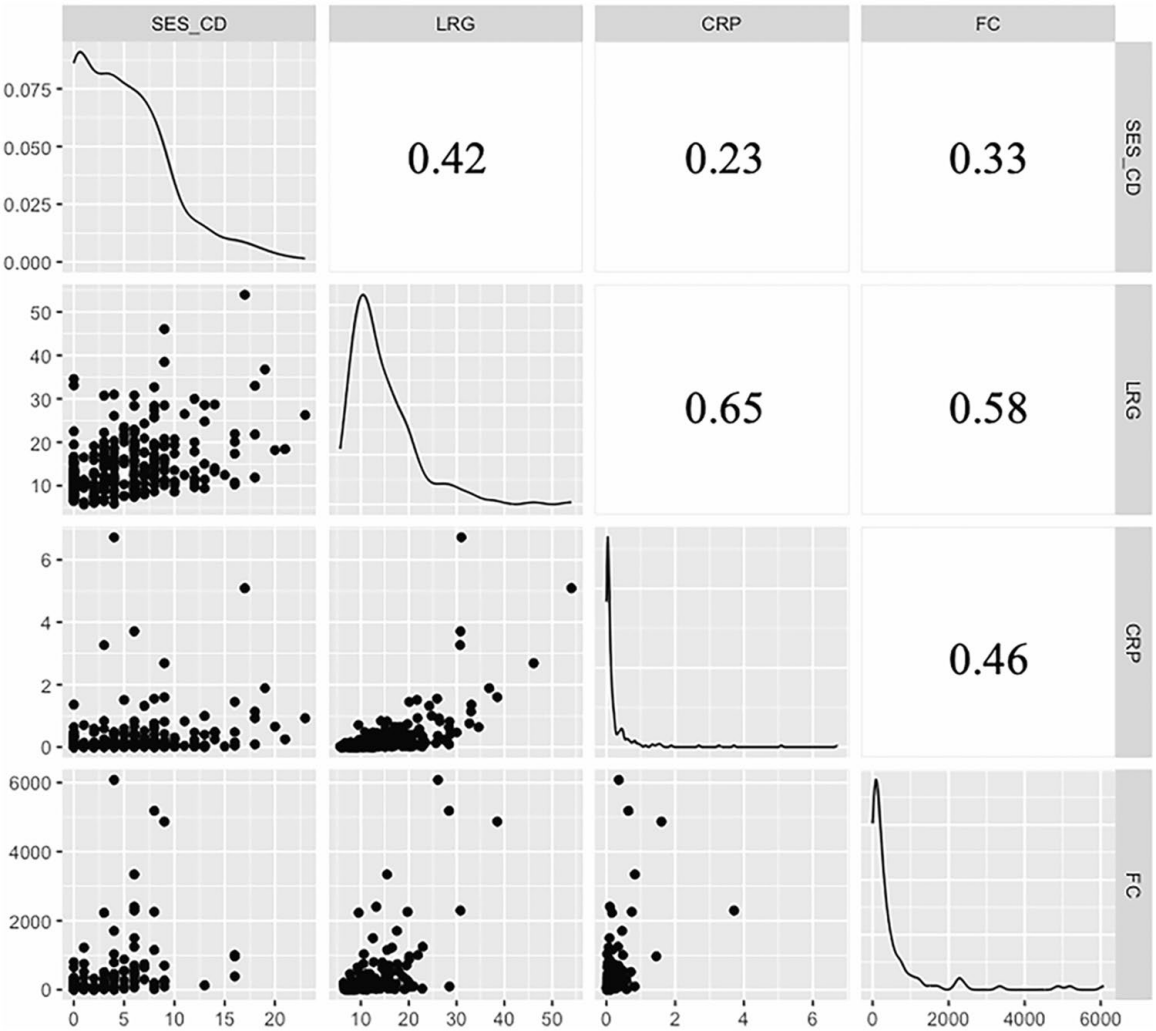
Scatter plots and correlation coefficients for LRG, CRP, FC, and modified SES-CD in SB patients. Each number represents a correlation coefficient. The correlation coefficients for LRG, CRP, FC, and modified SES-CD in SB patients were: 0.42 (95% CI 0.32–0.51) between modified SES-CD and LRG, 0.23 (95% CI 0.11–0.33) between modified SES-CD and CRP, 0.33 (0.16–0.48) between modified SES-CD and FC, 0.65 (0.58–0.71) between LRG and CRP, 0.58 (0.45–0.68) between LRG and FC, and 0.46 (0.31–0.58) between CRP and FC

The scatter plots and correlation coefficients for LRG, CRP, FC, and modified SES-CD in SB patients are shown in Fig. 1. The correlation coefficients for LRG, CRP, FC, and modified SES-CD in SB patients were: 0.42 (95% CI 0.32–0.51) between modified SES-CD and LRG, 0.23 (95% CI 0.11–0.33) between modified SES-CD and CRP, 0.33 (0.16–0.48) between modified SES-CD and FC, 0.65 (0.58–0.71) between LRG and CRP, 0.58 (0.45–0.68) between LRG and FC, and 0.46 (0.31–0.58) between CRP and FC. The scatter plots and correlation coefficients for LRG, CRP, FC, and modified SES-CD in SB and colon patients are shown in Supplementary Fig. 3. The correlation coefficients for LRG, CRP, FC, and modified SES-CD in SB and colon patients were as follows: 0.60 (95% CI 0.54–0.66) between modified SES-CD and LRG, 0.46 (95% CI 0.38–0.53) between modified SES-CD and CRP, 0.55 (95% CI 0.45–0.63) between modified SES-CD and FC, 0.73 (95% CI 0.69–0.77) between LRG and CRP, 0.59 (95% CI 0.50–0.67) between LRG and FC, and 0.37 (95% CI 0.25–0.48) between CRP and FC.

### Diagnostic accuracy in SB patients

**Fig. 2:**
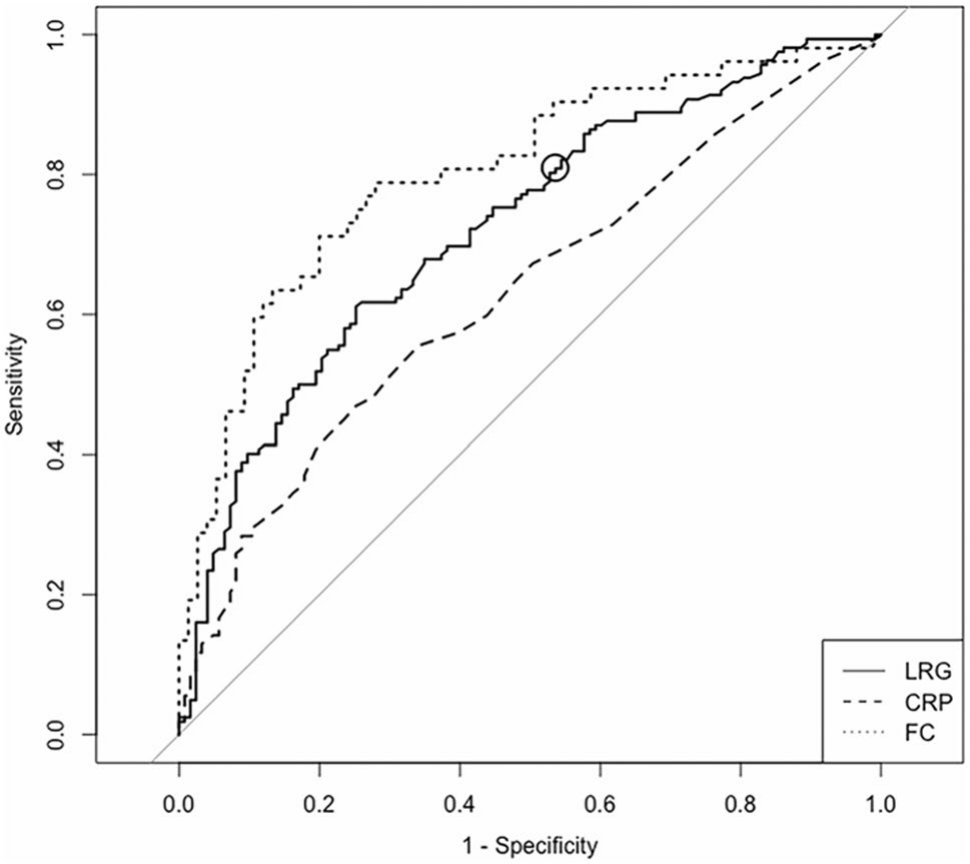
ROC curves of LRG, CRP, and FC for SB patients in diagnosing endoscopic remission. The ROC curves of LRG (solid line), CRP (dashed line), and FC (dotted line) are shown. The solid circle indicates the cut-off value of LRG. The AUCs (95%CI) of LRG, CRP, and FC were 0.72 (0.67–0.78), 0.63 (0.57–0.70), and 0.80 (0.72–0.88), respectively. A cut-off value of 10.5 for LRG had a sensitivity of 80.2% and specificity of 47.2%

The ROC curve of LRG for diagnosing endoscopic remission in SB patients yielded an AUC of 0.72 (95% CI 0.67–0.78), and a cut-off value of 10.5 had a sensitivity of 80.2% and specificity of 47.2% (Fig. 2). Meanwhile, CRP and FC had an AUC of 0.63 (0.57–0.70) and 0.80 (0.72–0.88), respectively. LRG had a significantly higher diagnostic accuracy versus CRP (*P* < 0.01), while there was no significant difference between LRG and FC (*P* = 0.12).

**Fig. 3:**
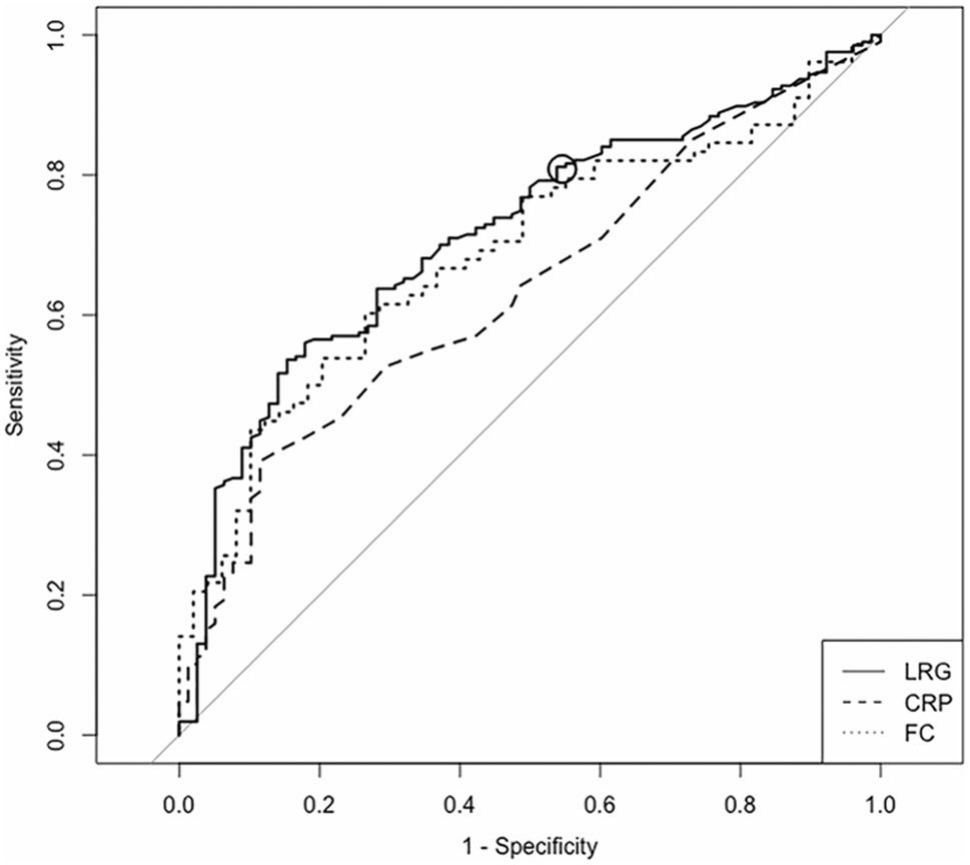
ROC curves of LRG, CRP, and FC for SB patients in diagnosing complete ulcer healing. The ROC curves of LRG (solid line), CRP (dashed line), and FC (dotted line) are shown. The solid circle indicates the cut-off value of LRG. The AUCs (95%CI) of LRG, CRP, and FC were 0.72 (0.65–0.78), 0.64 (0.57–0.70), and 0.69 (0.60–0.78), respectively. A cut-off value of 10.1 for LRG had a sensitivity of 81.2% and specificity of 46.2%

The ROC curve of LRG for diagnosing complete ulcer healing in SB patients yielded an AUC of 0.72 (0.65–0.78), and a cut-off value of 10.1 had a sensitivity of 81.2% and specificity of 46.2% (Fig. 3). Meanwhile, CRP and FC had an AUC of 0.64 (0.57–0.70) and 0.69 (0.60–0.78), respectively. LRG had a significantly higher diagnostic accuracy versus CRP (*P* < 0.01), while there was no significant difference between LRG and FC (*P* = 0.63).

### Diagnostic accuracy in SB and colon patients

**Fig. 4:**
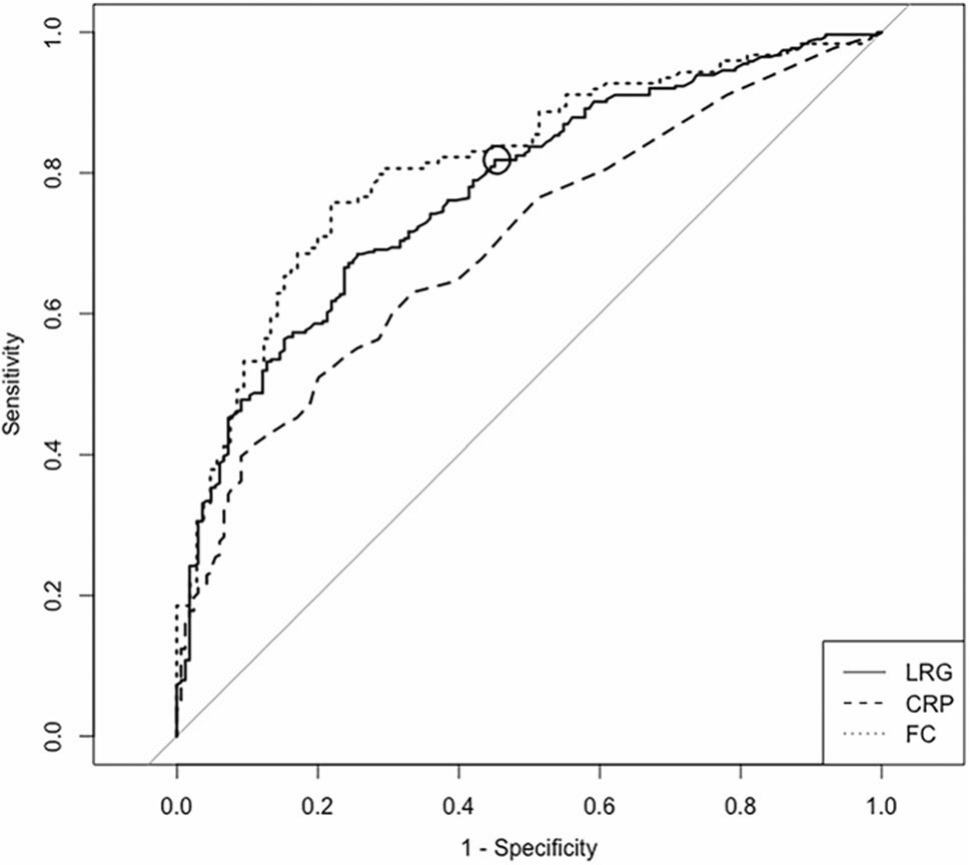
ROC curves of LRG, CRP, and FC for SB and colon patients in diagnosing endoscopic remission. The ROC curves of LRG (solid line), CRP (dashed line), and FC (dotted line) are shown. The solid circle indicates the cut-off value of LRG. The AUCs (95% CI) of LRG, CRP, and FC were 0.77 (0.73–0.82), 0.70 (0.65–0.75), and 0.81 (0.75–0.87), respectively. A cut-off value of 11.1 for LRG had a sensitivity of 81.8% and specificity of 54.9%

The ROC curve of LRG for diagnosing endoscopic remission in SB and colon patients yielded an AUC of 0.77 (95% CI 0.73–0.82), and a cut-off value of 11.1 had a sensitivity of 81.8% and specificity of 54.9% (Fig. 4). Meanwhile, CRP and FC had an AUC of 0.70 (0.65–0.75) and 0.81 (0.75–0.87), respectively. LRG had a significantly higher diagnostic accuracy versus CRP (*P* < 0.01), while there was no significant difference between LRG and FC (*P* = 0.30).

**Fig. 5:**
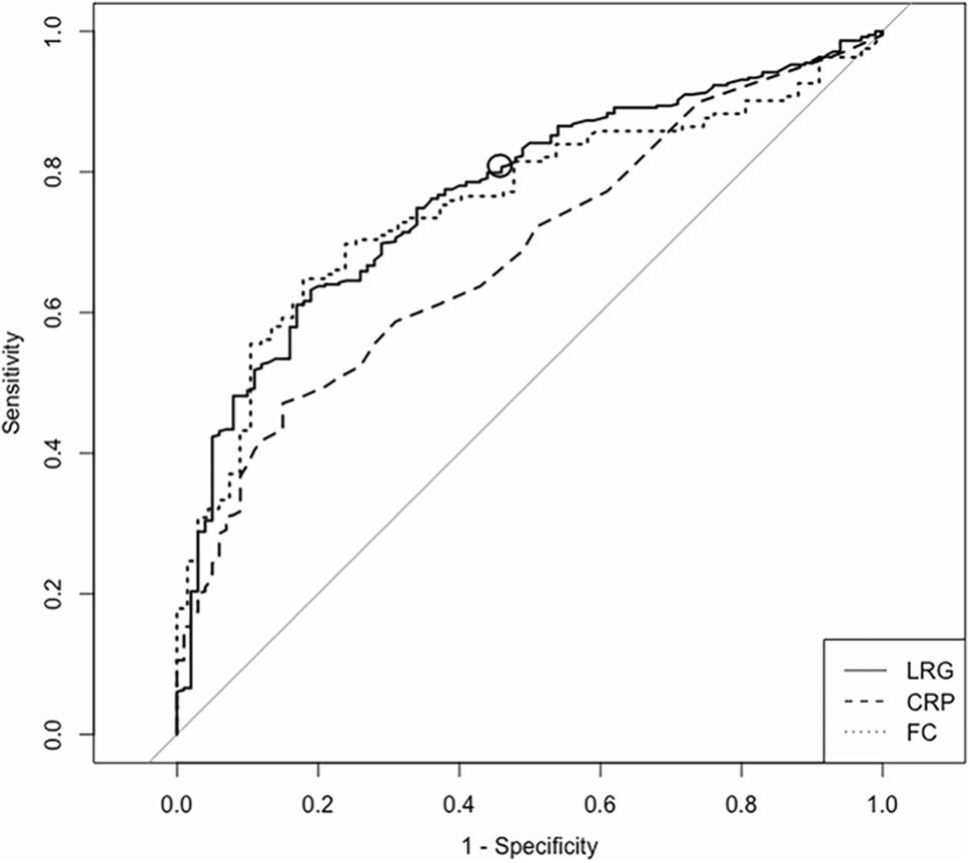
ROC curves of LRG, CRP, and FC for SB and colon patients in diagnosing complete ulcer healing. The ROC curves of LRG (solid line), CRP (dashed line), and FC (dotted line) are shown. The solid circle indicates the cut-off value of LRG. The AUCs (95% CI) of LRG, CRP, and FC were 0.77 (0.72–0.81), 0.68 (0.63–0.74), and 0.75 (0.69–0.82), respectively. A cut-off value of 10.6 for LRG had a sensitivity of 80.7% and specificity of 54.0%

The ROC curve of LRG for diagnosing complete ulcer healing in SB and colon patients yielded an AUC of 0.77 (0.72–0.81), and a cut-off value of 10.6 had a sensitivity of 80.7% and specificity of 54.0% (Fig. 5). Meanwhile, CRP and FC had an AUC of 0.68 (0.63–0.74) and 0.75 (0.69–0.82), respectively. LRG had a significantly higher diagnostic accuracy versus CRP (*P* < 0.01), while there was no significant difference between LRG and FC (*P* = 0.74).

The cut-off values, sensitivities, specificities, and AUCs across the different target populations and types of endoscopic activity are summarized in Table 2. The assumption of having no strong factors related to CD activity (which allows for ROC analysis without stratification by any factor) across all analyses was also confirmed (see Section S3 in Supplementary Materials). Furthermore, upon evaluating factors related to endoscopic activity based on a penalized logistic regression analysis with ridge penalty of the dichotomized modified SES-CD using many characteristics and outcome variables as covariates, LRG was shown to be the strongest factor in all analyses (see Section S4 in Supplementary Materials).

**Table 2:** Summary of cut-off values, sensitivities, specificities, and AUCs across all analyses

Target population	Endoscopic activity	Cut-off value	Sensitivity	Specificity	AUC
SB patients	Endoscopic remission	10.5	80.2%	47.2%	0.72
	Complete ulcer healing	10.1	81.2%	46.2%	0.72
SB and colon patients	Endoscopic remission	11.1	81.8%	54.9%	0.77
	Complete ulcer healing	10.6	80.7%	54.0%	0.77

## Discussion

We conducted a retrospective, multi-center study of CD patients undergoing retrograde BAE to determine the diagnostic accuracy and cut-off values of LRG in diagnosing CD activity in the SB. In diagnosing endoscopic remission, a cut-off value of 10.5 had a sensitivity of 80.2% and specificity of 47.2%. And, a cut-off value of 10.1 had a sensitivity of 81.2% and specificity of 46.2% for complete ulcer healing. LRG can compensate for at least two of the drawbacks of FC, which are the large within-sample variability and the psychological burden associated with submitting stool samples. Considering the advantages of LRG, especially its ease of implementation with a simple blood test, it can potentially be a useful triage marker for diagnosing SB-CD activity.

We believe some discussion of the calculated cut-off values is warranted. The difference in LRG cut-off values was only 0.4 in SB patients between the definitions of complete ulcer healing (modified SES-CD ≤ 1) and endoscopic remission (modified SES-CD ≤ 3) as endoscopic inactivity. This outcome appears to be influenced by the distribution shapes of LRG values and the numbers of patients with modified SES-CD scores of 2 and 3 (see Section S7 in Supplementary materials). Given that previous studies [1, 2] have shown no significant difference in clinical remission rates when the SES-CD score is below 5, the current findings may not be coincidental. Future prospective validation studies should consider defining endoscopic inactivity as either complete ulcer healing or endoscopic remission, rather than both. In addition, since CD patients with stricturing behavior accounted for 61.4% (175/285) of all participants, it is necessary to consider whether BAE via the retrograde approach alone was sufficient to observe the entire SB. As noted in the exclusion criteria, patients with severe SB strictures were excluded in advance. Of the patients, 61.4% had stricturing behavior, but many of the strictures had been removed by previous surgical procedures. In fact, among the 175 patients, the terminal ileum was observed in all cases, and the proximal ileum in 168 cases as expected. To assess full SB, BAE via not only the retrograde approach but also antegrade approach are required. In our study, only 29 patients underwent jejunal observation via the antegrade approach. However, CT or small bowel follow-through confirmed the absence of highly active lesions in most cases. Furthermore, the participating hospitals, which are tertiary care institutions, treat a high volume of CD patients, and we believe that the major SB lesions can be sufficiently assessed using the retrograde approach, taking into account complementary information from other modalities.

To validate the LRG cut-off values and diagnostic accuracy identified in this study, we compared our findings with eight clinical trials involving participants who underwent ileocolonoscopy or BAE [14–21]. Reported LRG cut-off values ranged from 8.9 to 14.3 μg/mL (see Table S1, Supplementary Materials), but variability in endoscopic scoring systems, definitions of endoscopic inactivity, and target populations limited direct comparisons (see Table S2 in Supplementary Materials). Among three studies that measured LRG and conducted BAE on most patients [14, 16, 17], cut-off values of 13, 13.4, and 8.9 μg/mL were reported. Our study recruited patients who underwent LRG and BAE within 30 days, while two studies had 8-week intervals [14, 17], and one had a 2-week interval [16]. Although these differences could influence LRG cut-off values, sensitivity, and specificity, variations in patient characteristics and study designs made it challenging to pinpoint the reasons for discrepancies in diagnostic accuracy and cut-off values for LRG.

Notably, this study observed lower specificity for LRG, as optimizing sensitivity (targeted at 80%) reduced specificity, which still remained lower than in previous studies. For FC, targeting 80% sensitivity yielded specificities of 62.7% for endoscopic remission and 40.8% for complete ulcer healing in this study; while FC specificity exceeded LRG for endoscopic remission, it was lower for complete ulcer healing. Meta-analyses by Rokkas et al. [5] and Cannatelli et al. [6] reported higher FC specificities (72.1% and 84.4%, respectively), likely reflecting differences in patient populations or study designs. These results highlight the influence of methodological variations and patient characteristics on diagnostic performance, and a prospective study is planned to validate these findings further.

We believe that LRG cut-off values can effectively detect the possibility of SB lesions. In this study, BAE was used to assess these lesions. However, it is important to note that BAE is an invasive procedure, whereas other imaging options for evaluating the SB include capsule endoscopy (CE) and MR enterography (MRE). CE offers a non-invasive way to assess luminal CD throughout the SB, although the definition of mucosal healing remains unclear. Its use is limited to patients without strictures due to the risk of capsule retention, which restricts its routine use in CD. Conversely, MRE provides a comprehensive view of the entire abdomen but primarily detects extraintestinal lesions and is less sensitive to luminal abnormalities. The goal of achieving transmural remission presents a clinical challenge for future management. Nevertheless, BAE remains a valuable option for patients with suspected SB involvement based on elevated LRG levels. The additional advantages of BAE include the ability to obtain histological samples and perform therapeutic procedures, such as endoscopic balloon dilation. In routine clinical practice, it is essential to carefully consider the pros and cons of each modality when evaluating the SB of CD patients.

The main limitation of our study is that the study cohort may have included many CD patients with having a refractory course and high disease activity due to the involvement of primarily higher tertiary care institutions. In addition, retrospective analysis of CD patients who underwent both LRG measurement and BAE could have led to a larger number of severely ill patients. However, although the median duration of illness was 11 yr, 154 (54%) patients had a history of bowel surgery, and 221 (77.5%) had a history of bowel complications, the median CDAI was 63, median LRG was 12.6 μg/mL, and median CRP was 0.1 mg/dL, which were not high. Thus, despite many of the patients having a refractory course, their disease activity was not elevated. In other words, the presence of selection bias regarding severity was expected to be a limitation of our study, but in fact, contrary to the expectation, the number of severely ill patients was not high in this study. The other limitation of our study is that the modified SES-CD score was determined by a single endoscopist at each facility but not a central assessment committee. To ensure rigor, the modified SES-CD score should have been validated by multiple diagnosticians reviewing the endoscopic images. However, it is worth noting that a meta-analysis has pointed out that the inter-observer agreement of SED-CD score ranged from moderate to good [22].

Few papers have scrutinized SB lesions in depth and evaluated the diagnostic accuracy of biomarkers in CD patients. This is why we could obtain highly stable cut-off values. Our findings suggest that the use of the cut-off values with high sensitivity levels for SB lesions may minimize the need for invasive BAE or lengthy capsule endoscopy, and can lead to more practical and smooth monitoring in T2T strategy [23]. A prospective study planned by our research group will validate the accuracy of LRG testing using these cut-off values; this can determine the clinical utility of LRG compared to FC for diagnosing CD activity.

## Supplementary Information

Below is the link to the electronic supplementary material.

Supplementary file1 (DOCX 3841 KB)

## References

[1] Takenaka K, Ohtsuka K, Kitazume Y, et al. Utility of magnetic resonance enterography for small bowel endoscopic healing in patients with Crohn’s disease. Am J Gastroenterol. 2018;113(2):283–94. 29257147 10.1038/ajg.2017.464

[2] Takabayashi K, Hosoe N, Kato M, et al. Significance of endoscopic deep small bowel evaluation using balloon-assisted enteroscopy for Crohn’s disease in clinical remission. J Gastroenterol. 2021;56(1):25–33. 33078323 10.1007/s00535-020-01737-0

[3] Möschler O, May A, Müller MK, et al. Complications in and performance of double-balloon enteroscopy (DBE): results from a large prospective DBE database in Germany. Endoscopy. 2011;43(6):484–9. 21370220 10.1055/s-0030-1256249

[4] Bossuyt PM, Irwig L, Craig J, et al. Comparative accuracy: assessing new tests against existing diagnostic pathways. BMJ. 2006;332(7549):1089–92. 16675820 10.1136/bmj.332.7549.1089 PMC1458557

[5] Rokkas T, Portincasa P, Koutroubakis IE. Fecal calprotectin in assessing inflammatory bowel disease endoscopic activity: a diagnostic accuracy meta-analysis. J Gastrointest Liver Dis. 2018;27(3):299–306. 10.15403/jgld.2014.1121.273.pti 30240474

[6] Cannatelli R, Bazarova A, Zardo D, et al. Fecal calprotectin thresholds to predict endoscopic remission using advanced optical enhancement techniques and histological remission in IBD patients. Inflamm Bowel Dis. 2021;27(5):647–54. 32592477 10.1093/ibd/izaa163

[7] Du L, Foshaug R, Huang VW, et al. Within-stool and within-day sample variability of fecal calprotectin in patients with inflammatory bowel disease: a prospective observational study. J Clin Gastroenterol. 2018;52(3):235–40. 28009684 10.1097/MCG.0000000000000776

[8] Joshi S, Lewis SJ, Creanor S, et al. Age-related faecal calprotectin, lactoferrin and tumour M2-PK concentrations in healthy volunteers. Ann Clin Biochem. 2010;47(3):259–63. 19740914 10.1258/acb.2009.009061

[9] Zittan E, Gralnek IM, Berns MS. The new proactive approach and precision medicine in Crohn’s disease. Biomedicines. 2020;8(7):193. 32635316 10.3390/biomedicines8070193 PMC7400127

[10] Haupt H, Baudner S. Isolierung und Charakterisierung eines bisher unbekannten leucinreichen 3.1S-alpha2-Glykoproteins aus Humanserum [Isolation and characterization of an unknown, leucine-rich 3.1-S-alpha2-glycoprotein from human serum (author’s transl)]. Hoppe Seylers Z Physiol Chem. 1977;358(6):639–46. 69600

[11] Okita M, Nakashima K, Yamamura T, et al. Systematic review and meta-analysis of the use of serum leucine-rich Alpha-2 glycoprotein to assess Crohn’s disease activity. Inflamm Bowel Dis. 2024;30(5):780–7. 37506169 10.1093/ibd/izad128

[12] Takenaka K, Ohtsuka K, Kitazume Y, et al. Correlation of the endoscopic and magnetic resonance scoring systems in the deep small intestine in Crohn’s disease. Inflamm Bowel Dis. 2015;21(8):1832–8. 26020602 10.1097/MIB.0000000000000449

[13] Takenaka K, Kawamoto A, Hibiya S, et al. Higher concentrations of cytokine blockers are needed to obtain small bowel mucosal healing during maintenance therapy in Crohn’s disease. Aliment Pharmacol Ther. 2021;54(8):1052–60. 34323301 10.1111/apt.16551

[14] Kawamoto A, Takenaka K, Hibiya S, et al. Combination of leucine-rich alpha-2 glycoprotein and fecal markers detect Crohn’s disease activity confirmed by balloon-assisted enteroscopy. Intest Res. 2024;22(1):65–74. 37939721 10.5217/ir.2023.00092 PMC10850704

[15] Matsumoto S, Mashima H. Usefulness of serum leucine-rich alpha 2 glycoprotein in Crohn’s disease is there any difference between small intestine and colonic lesions? Crohns Colitis 360. 2023;5(3):028. 10.1093/crocol/otad028 PMC10243872 37288327

[16] Kawamura T, Yamamura T, Nakamura M, et al. Accuracy of serum leucine-rich alpha-2 glycoprotein in evaluating endoscopic disease activity in Crohn’s disease. Inflamm Bowel Dis. 2023;29(2):245–53. 35436345 10.1093/ibd/izac076

[17] Kawamoto A, Takenaka K, Hibiya S, Ohtsuka K, Okamoto R, Watanabe M. Serum leucine-rich α_2_glycoprotein: a novel biomarker for small bowel mucosal activity in Crohn’s disease. Clin Gastroenterol Hepatol. 2022;20(5):e1196–200. 34216822 10.1016/j.cgh.2021.06.036

[18] Yoshida T, Shimodaira Y, Fukuda S, et al. Leucine-rich alpha-2 glycoprotein in monitoring disease activity and intestinal stenosis in inflammatory bowel disease. Tohoku J Exp Med. 2022;257(4):301–8. 35598974 10.1620/tjem.2022.J042

[19] Abe I, Shiga H, Chiba H, et al. Serum leucine-rich alpha-2 glycoprotein as a predictive factor of endoscopic remission in Crohn’s disease. J Gastroenterol Hepatol. 2022;37(9):1741–8. 35641439 10.1111/jgh.15907

[20] Shimoyama T, Yamamoto T, Yoshiyama S, Nishikawa R, Umegae S. Leucine-rich alpha-2 glycoprotein is a reliable serum biomarker for evaluating clinical and endoscopic disease Activity in inflammatory bowel disease. Inflamm Bowel Dis. 2023;29(9):1399–408. 36334015 10.1093/ibd/izac230

[21] Yasutomi E, Inokuchi T, Hiraoka S, et al. Leucine-rich alpha-2 glycoprotein as a marker of mucosal healing in inflammatory bowel disease. Sci Rep. 2021;11(1):11086. 34045529 10.1038/s41598-021-90441-x PMC8160157

[22] Hashash JG, Yu Ci Ng F, Farraye FA, et al. Inter- and intraobserver variability on endoscopic scoring systems in Crohn’s disease and ulcerative colitis: a systematic review and meta-analysis. Inflamm Bowel Dis. 2024;30(11):2217–26. 38547325 10.1093/ibd/izae051

[23] Turner D, Ricciuto A, Lewis A, et al. STRIDE-II: an update on the selecting therapeutic targets in inflammatory bowel disease (STRIDE) initiative of the international organization for the study of IBD (IOIBD): determining therapeutic goals for treat-to-target strategies in IBD. Gastroenterology. 2021;160(5):1570–83. 33359090 10.1053/j.gastro.2020.12.031

