# Supplementary material for: Diagnostic accuracy and cut-off values of serum leucine-rich alpha-2 glycoprotein for Crohn’s disease activity in the small bowel
Source: J Gastroenterol. 2025 Feb 14;60(5):573–82. doi: 10.1007/s00535-025-02223-1 (PMC12014797; doi:10.1007/s00535-025-02223-1)
Supplement: Supplementary file 1 — Supplementary file1 (DOCX 3841 KB) [file 535_2025_2223_MOESM1_ESM.docx]

Supplementary materials

Section S1. Boxplots and scatter plots of modified SES-CD


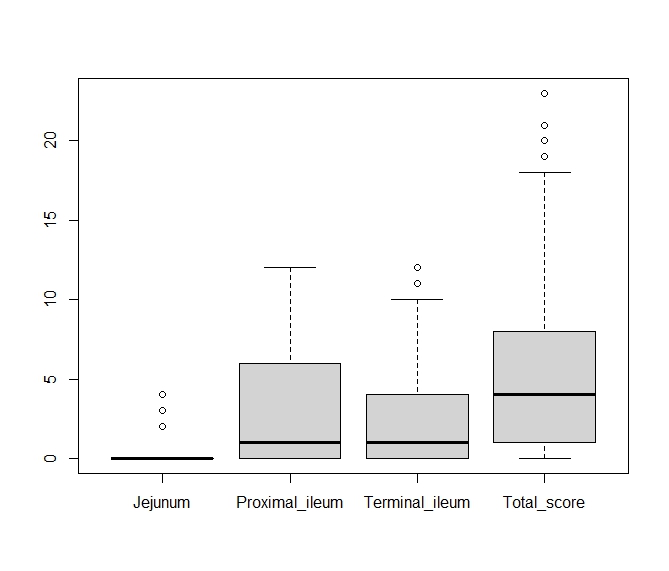


Supplementary Figure S1. Boxplots of modified SES-CD in SB patients.

The median (IQR) for each site in SB patients was as follows: 0 (0–0) in the jejunum, 1 (0–6) in the proximal ileum, and 1 (0–4) in the terminal ileum. The median (IQR) of the total modified SES-CD score was 4 (1–8).


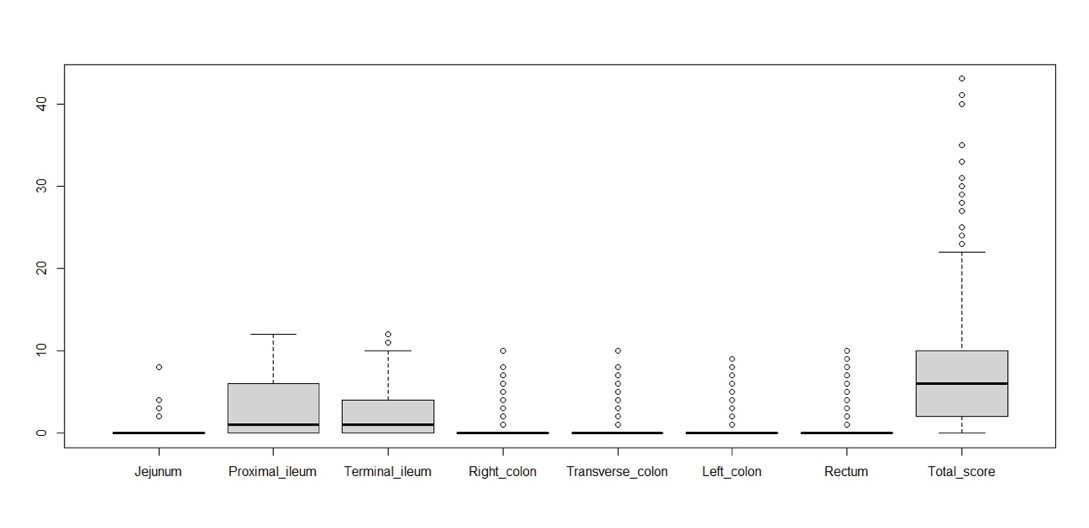


Supplementary Figure S2. Boxplots of modified SES-CD in SB and colon patients.

The median (IQR) for each site in SB and colon patients was as follows: 0 (0–0) in the jejunum, 1 (0–6) in the proximal ileum, 1 (0–4) in the terminal ileum, 0 (0–0) in the right colon,0 (0–0) in the transverse colon, 0 (0–0) in the left colon, and 0 (0–0) in the rectum. The median (IQR) of total modified SES-CD score was 6 (2–10).


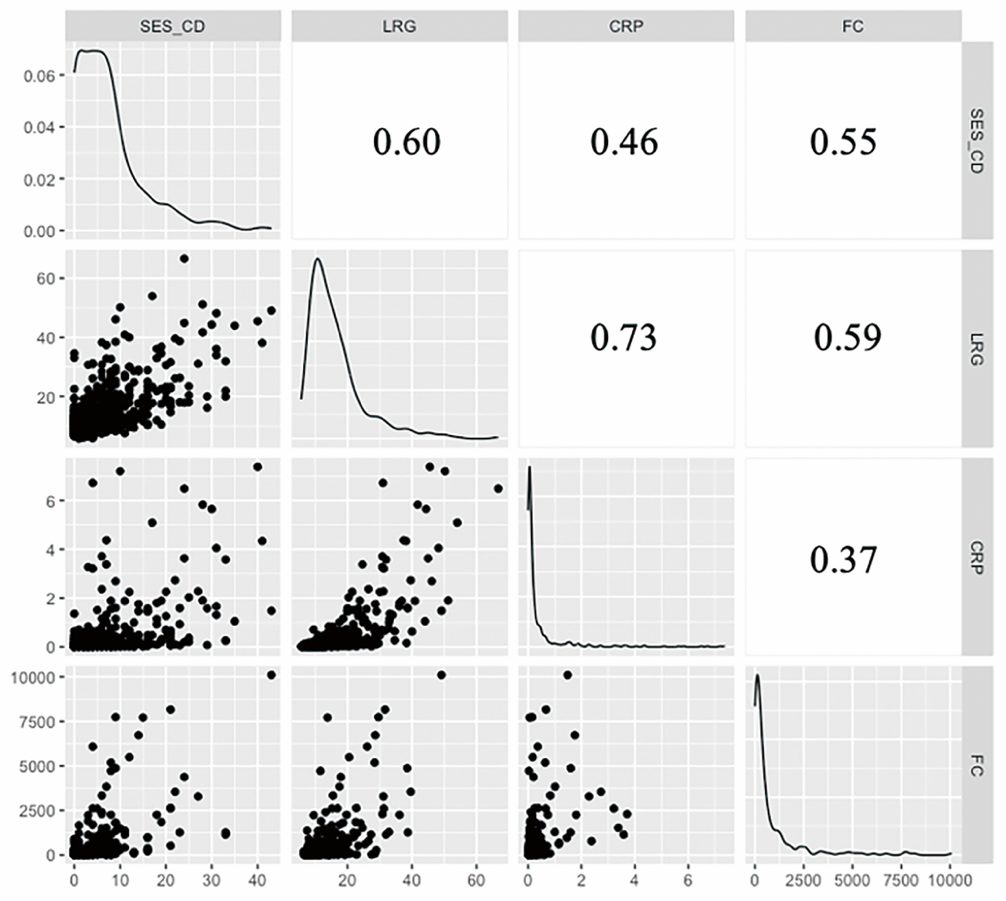


Supplementary Figure S3. Scatter plots and correlation coefficients of LRG, CRP, FC, and modified SES-CD in SB and colon patients.

Scatter plots and correlation coefficients for LRG, CRP, FC, and modified SES-CD in SB and colon patients. Each number represents a correlation coefficient. The correlation coefficients for LRG, CRP, FC, and modified SES-CD in SB and colon patients were as follows: 0.60 (95%CI: 0.54–0.66) between modified SES-CD and LRG, 0.46 (95%CI: 0.38–0.53) between modified SES-CD and CRP, 0.55 (95%CI: 0.45–0.63) between modified SES-CD and FC, 0.73 (95%CI: 0.69–0.77) between LRG and CRP, 0.59 (95%CI: 0.50–0.67) between LRG and FC, and 0.37 (95%CI: 0.25–0.48) between CRP and FC.

Section S2. Methods of additional analyses

1. Additional analysis #1: confirming that the ROC curve of LRG can be estimated without stratification by any factor.

We conducted multivariate logistic regression analysis with the dichotomized modified SES-CD as the objective variable and the following clinical factors as explanatory variables: age, sex, CDAI, location, behavior, disease duration, history of major intestinal surgery, fistula, complications of other immunological diseases, complication of liver cirrhosis, smoking (i.e., current smoker, previous smoker, or non-smoker), anal disease (i.e., perianal abscess or anal fistula), non-steroidal anti-inflammatory drugs, steroids, antiplatelet drugs, immunomodulators, infliximab, adalimumab, vedolizumab, ustekinumab, risankizumab, and upadacitinib. For the estimated logistic regression model, the predicted probability (score) that each patient would have an outcome of 1 was calculated. Based on the scores obtained on multivariate logistic regression analysis (0–1 point), we stratified patients into low-, middle-, and high-score groups such that the number of patients in the three strata were almost same, then the ROC curve of LRG was estimated in each stratum. If the three ROC curves do not change significantly, then we determined that there was no need to adjust the LRG cut-off value for each factor.

2. Additional analysis #2: determining which single factor has the greatest impact on endoscopic activity

We conducted penalized logistic regression analysis (Ridge penalty) with the dichotomized modified SES-CD as the objective variable and the following factors plus the aforementioned clinical factors as explanatory variables: LRG, CRP, Alb, AST, ALT and Hb (FC was not included due to the large amount of missing data). The prediction accuracy of the generated prediction scores was evaluated via 5-fold cross validation. For the confidence intervals of each regression coefficient and AUC, the boot-strap method was repeated 1000 times, and the 95% confidence intervals were defined between the lower 2.5% and 97.5% points using the percentile method. After standardizing all explanatory variables including binary variables, regression analysis was performed to compare the effect sizes for each factor.

3. Additional analysis #3: evaluating the diagnostic accuracy of LRG and FC in combination.

We conducted multivariate logistic regression analysis with the dichotomized modified SES-CD as the objective variable and LRG and FC as explanatory variables, and the ROC curve was estimated. The prediction accuracy of the generated prediction scores (AUC) was evaluated by 5-fold cross validation. For the confidence intervals of AUC, the boot-strap method was repeated 1000 times, and the 95% confidence intervals were defined between the lower 2.5% and 97.5% points by the percentile method.

4. Additional analysis #4: determining the diagnostic accuracy of LRG stratified with complications of anal disease.

Since the association between complications of anal disease (i.e., perianal abscess or anal fistula) and LRG has been suggested by some researchers [1], ROC curves stratified by complication of anal disease were estimated.

Section S3. Results of additional analyses #1

Results of additional analysis #1: diagnosing endoscopic remission in SB patients

The cut-off values of scores for the low- and middle-score groups and for the middle- and high-score groups were 0.426 and 0.6326, respectively. The ROC curves for each strata are shown in Supplementary Figure S4. A comparison of the ROC curves between the low- and middle-score groups and between the middle- and high-score groups by Delong’s two-tailed test yielded P values of 0.24 and 0.97, respectively; neither result was significant.


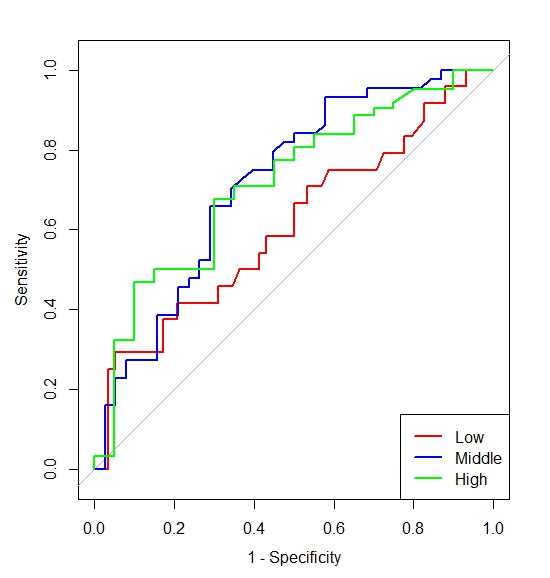


Supplementary Figure S4. ROC curves of LRG with stratification of logistic scores in diagnosing endoscopic remission in SB patients.

Results of additional analysis #1: diagnosing complete ulcer healing in SB patients

The cut-off values of scores for the low- and middle-score groups and for the middle- and high-score groups were 0.627 and 0.807, respectively. The ROC curves for each strata are shown in Supplementary Figure S5. A comparison of the ROC curves between the low- and middle-score groups and between the middle- and high-score groups by Delong’s two-tailed test yielded P values of 0.49 and 0.61 respectively; neither result was significant.


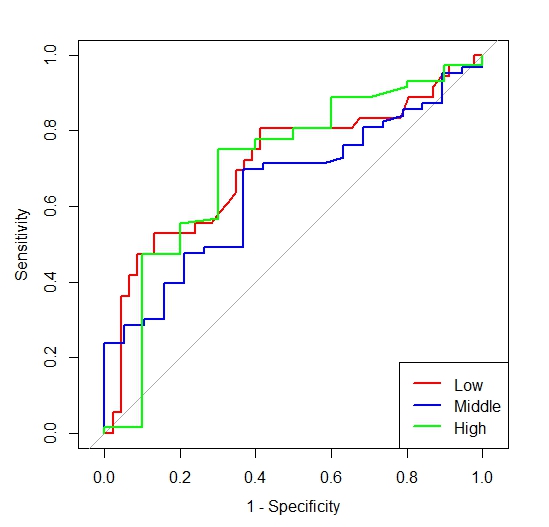


Supplementary Figure S5. ROC curves of LRG with stratification of logistic scores in diagnosing complete ulcer healing in SB patients.

Results of additional analysis #1: diagnosing endoscopic remission in SB and colon patients

The cut-off values of scores for the low- and middle-score groups and for the middle- and high-score groups were 0.549 and 0.75, respectively. The ROC curves for each strata are shown in Supplementary Figure S6. A comparison of the ROC curves between the low- and middle-score groups and between the middle- and high-score groups by Delong’s two-tailed test yielded P values of 0.77 and 0.51, respectively; neither result was significant.


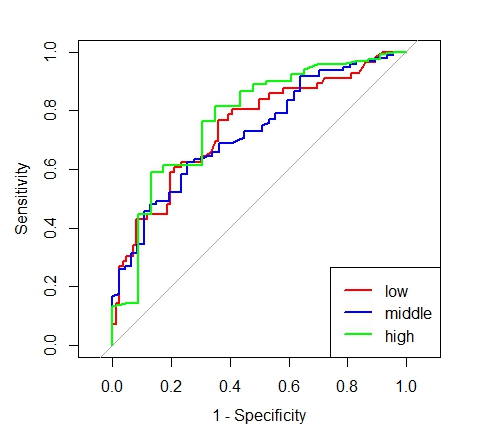


Supplementary Figure S6. ROC curves of LRG with stratification of logistic scores in diagnosing endoscopic remission in SB and colon patients.

Results of additional analysis #1: diagnosing complete ulcer healing in SB and colon patients.

The cut-off values of scores for the low- and middle-score groups and for the middle- and high-score groups were 0.725 and 0.881, respectively. The ROC curves for each strata are shown in Supplementary Figure S7. A comparison of the ROC curves between the low- and middle-score groups and between the middle- and high-score groups by Delong’s two-tailed test yielded P values of 0.55 and 0.41, respectively; neither result was significant.


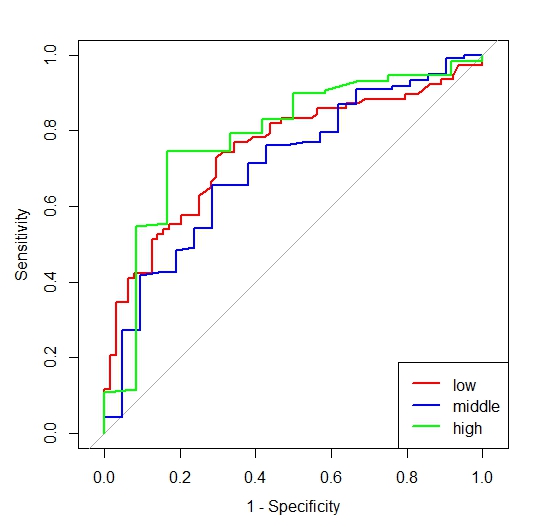


Supplementary Figure S7. ROC curves of LRG with stratification of logistic scores in diagnosing complete ulcer healing in SB and colon patients.

Section S4. Results of additional analysis #2

Results of additional analysis #2: diagnosing endoscopic remission in SB patients

The point estimate (95% confidence interval) for each variable is shown as follows:

Age: 0.10 (−0.02–0.37), sex 0.05 (−0.09–0.35), CDAI: 0.14 (0.04–0.55), fistula: 0.12 (0.06–0.44), location (SB & colon): −0.06 (−0.30–0.11), Behavior B2: 0.13 (0.05–0.61), Behavior B3: 0.03 (−0.10–0.36), duration: −0.03 (−0.37–0.09), previous surgery: 0.01 (−0.27 o 0.16), previous smoker: 0.05 (−0.12–0.37), current smoker: 0.02 (−0.11–0.30), steroid: 0.03(−0.10–0.32), IM: −0.05 (−0.27–0.15), IFX: −0.05 (−0.32–0.09), ADA: −0.02 (−0.24–0.16), VDZ: 0.04 (−0.22–0.32), UST: −0.02 (−0.27–0.15), RIS –0.07 (−0.20–0.00), UPA: −0.06 (−0.21–0.0), NSAIDs: 0.01 (−0.10–0.19), LC: −0.06 (−0.26–0.0), Immune diseases: −0.06 (−0.55–0.17), anal involvement (non-active): 0.04 (−0.10–0.38), anal involvement (active): 0.02 (−0.17–0.27), LRG: 0.24 (0.18–0.92), CRP: 0.07 (−0.12–0.32), Alb: −0.12 (−0.43–0.02), AST: −0.00 (−0.15–0.25), ALT: −0.09 (−0.45–0.00), Hb: 0.00 (−0.10–0.45).

The variables whose 95% confidence intervals did not cross zero were CDAI, fistula, Behavior B2, and LRG, with LRG having the largest effect size. The point estimate (95% confidence interval) for AUC was 0.70 (95%CI: 0.67–0.82).

Results of additional analysis #2: diagnosing complete ulcer healing in SB patients

The point estimate (95% confidence interval) for each variable is shown as follows:

Age: 0.11 (−0.08–0.39), sex 0.10 (−0.12–0.42), CDAI: 0.15 (−0.03–0.54), fistula: 0.15 (0.05–0.44), location (SB & colon): −0.17 (−0.45–0.03), Behavior B2: 0.18 (0.03–0.65), Behavior B3: 0.07 (−0.08–0.47), duration: −0.04 (−0.32–0.19), previous surgery: −0.02 (−0.39 o 0.14), previous smoker: 0.13 (−0.04–0.56), current smoker: 0.04 (−0.14–0.36), steroid: −0.00(−0.20–0.30), IM: −0.07 (−0.29–0.17), IFX: −0.17 (−0.58––0.01), ADA: −0.04 (−0.34–0.14), VDZ: 0.05 (−0.25–0.40), UST: −0.14 (−0.54–0.02), RIS 0.06 (0.00–0.18), UPA: −0.11 (−0.27–0.00), NSAIDs: −0.01 (−0.12–0.14), LC: −0.11 (−0.28–0.00), Immune diseases: −0.04 (−0.34–0.16), anal involvement (non-active): 0.01 (−0.18–0.28), anal involvement (active): 0.00 (−0.26–0.33), LRG: 0.35 (0.16–1.13), CRP: 0.14 (0.03–0.59), Alb: −0.08 (−0.33–0.22), AST: −0.04 (−0.27–0.18), ALT: −0.14 (−0.50–0.02), Hb: 0.10 (−0.05–0.55).

The variables whose 95% confidence intervals did not cross zero were fistula, Behavior B2, LRG, CRP, and IFX, with LRG having the largest effect size. The point estimate (95% confidence interval) for AUC was 0.69 (95%CI: 0.67–0.84).

Results of additional analysis #2: diagnosing endoscopic remission in SB and colon patients

The point estimate (95% confidence interval) for each variable is shown as follows:

Age: −0.03 (−0.25–0.15), sex 0.14 (−0.03–0.41), CDAI: 0.26 (0.07–0.52), fistula: 0.26 (0.16–0.45), location (colon): −0.29 (−0.53–−0.15), location (SB & colon): 0.11 (−0.09–0.32), Behavior B2: 0.17 (−0.00–0.43), Behavior B3: 0.00 (−0.22–0.22), duration: −0.08 (−0.31–0.15), previous surgery: −0.12 (−0.41 o 0.07), previous smoker: 0.11 (−0.07–0.36), current smoker: 0.09 (−0.07–0.31), steroid: 0.10 (−0.06–0.33), IM: −0.13 (−0.34–0.04), IFX: −0.13 (−0.37–0.05), ADA: −0.02 (−0.24–0.16), VDZ: 0.08 (−0.15–0.34), UST: −0.08 (−0.31–0.10), RIS –0.22 (−0.40–0.01), UPA: −0.13 (−0.21–0.0), NSAIDs: 0.04 (−0.07–0.19), antiplatelet:0.06 (0.00–0.12), LC: −0.13 (−0.24–0.00), Immune diseases: −0.07 (−0.48–0.21), anal involvement (non-active): 0.13 (−0.02–0.36), anal involvement (active): 0.13 (−0.07–0.40), LRG: 0.71 (0.47–1.28), CRP: 0.25 (0.04–0.64), Alb: −0.26 (−0.58–−0.06), AST: 0.05 (−0.13–0.31), ALT: -0.11 (−0.40–0.08), Hb: −0.02 (−0.26–0.22).

The variables whose 95% confidence intervals did not cross zero were CDAI, fistula, location (colon), LRG, CRP, and Alb, with LRG having the largest effect size. The point estimate (95% confidence interval) for AUC was 0.79 (95%CI: 0.76–0.86).

Results of additional analysis #2: diagnosing complete ulcer healing in SB and colon patients

The point estimate (95% confidence interval) for each variable is shown as follows:

Age: −0.01 (−0.30–0.25), sex 0.12 (−0.10–0.44), CDAI: 0.24 (0.03–0.55), fistula: 0.20 (0.12–0.42), location (colon): −0.34 (−0.64–0.42), location (SB & colon): 0.01 (−0.24–0.24), Behavior B2: 0.16 (−0.02–0.51), Behavior B3: 0.11 (−0.08–0.44), duration: −0.12 (−0.41–0.16), previous surgery: −0.10 (−0.47 o 0.09), previous smoker: 0.25 (0.07–0.65), current smoker: 0.10 (−0.08–0.39), steroid: 0.00 (−0.20–0.27), IM: −0.16 (−0.42–0.03), IFX: −0.24 (−0.56–−0.05), ADA: −0.04 (−0.32–0.14), VDZ: 0.09 (−0.21–0.43), UST: −0.23 (−0.57–−0.06), RIS 0.09 (0.00–0.20), UPA: −0.15 (−0.26–0.0), NSAIDs: −0.01 (−0.10–0.14), antiplatelet:0.04 (0.00–0.09), LC: −0.15 (−0.29–0.0), Immune diseases: 0.04 (−0.18–0.32), anal involvement (non-active): 0.05 (−0.17–0.28), anal involvement (active): 0.05 (−0.28–0.38), LRG: 0.66 (0.40–1.49), CRP: 0.27 (0.16–0.66), Alb: −0.16 (−0.42–0.14), AST: −0.01 (−0.23–0.31), ALT: −0.15 (−0.50–0.05), Hb: 0.04 (−0.20–0.36).

The variables whose 95% confidence intervals did not cross zero were CDAI, fistula, previous smoker, IFX, UST, antiplatelet, LRG, and CRP, with LRG having the largest effect size. The point estimate (95% confidence interval) for AUC was 0.77 (95%CI: 0.74–0.87).

Section S5. Results of additional analysis #3

Results of additional analysis #3: diagnosing endoscopic remission in SB patients


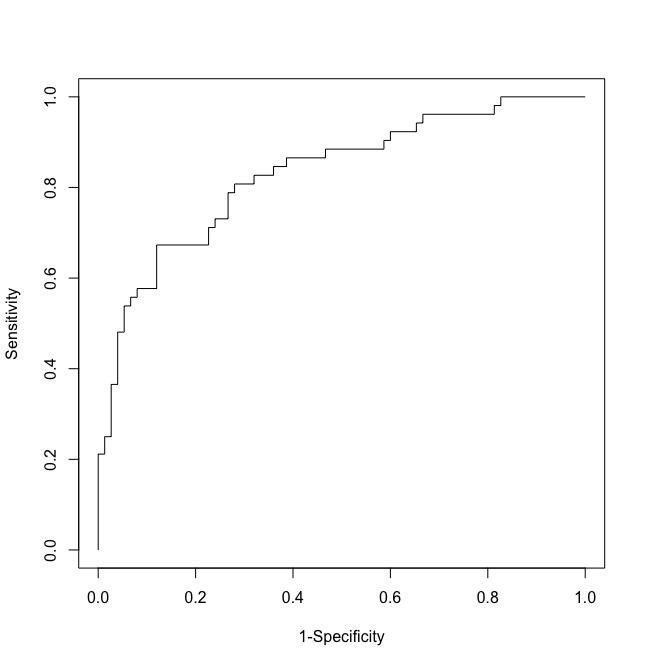


Supplementary Figure S8. ROC curve of the combination of LRG and FC in diagnosing endoscopic remission in SB patients.

AUC: 0.83

Point estimate of AUC and the 95% confidence interval obtained via cross validation and the boot-strap method: 0.83 (95%CI: 0.75–0.91).

Results of additional analysis #3: diagnosing complete ulcer healing in SB patients


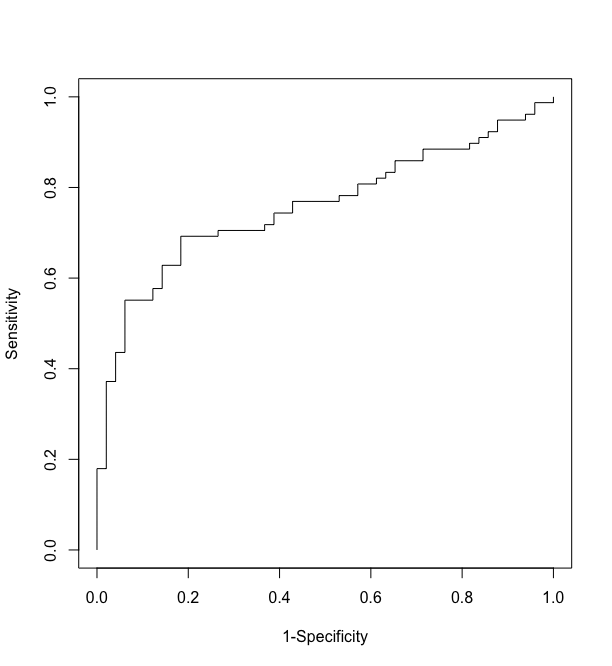


Supplementary Figure S9. ROC curve of the combination of LRG and FC in diagnosing complete ulcer healing in SB patients.

AUC: 0.76

Point estimate of AUC and the 95% confidence interval obtained via cross validation and the boot-strap method: 0.75 (95%CI: 0.67–0.84).

Results of additional analysis #3: diagnosing endoscopic remission in SB and colon patients


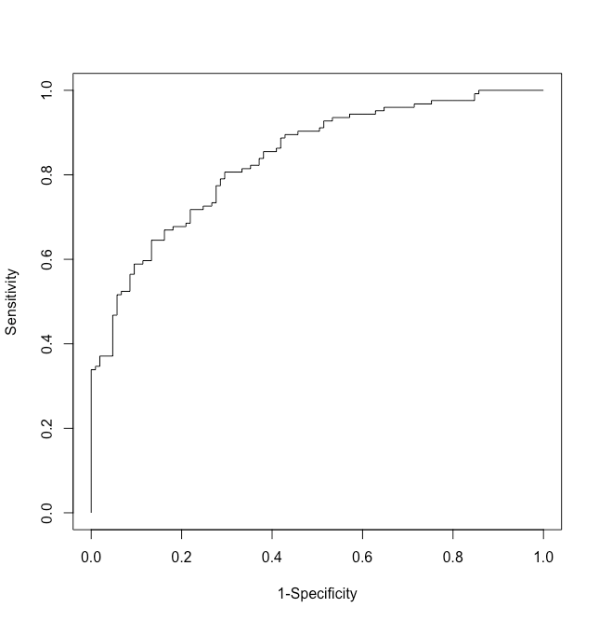


Supplementary Figure S10. ROC curve of the combination of LRG and FC in diagnosing endoscopic remission in SB and colon patients.

AUC: 0.84

Point estimate of AUC and the 95% confidence interval obtained via cross validation and the boot-strap method: 0.83 (95%CI: 0.78–0.89).

Results of additional analysis #3: diagnosing complete ulcer healing in SB and colon patients


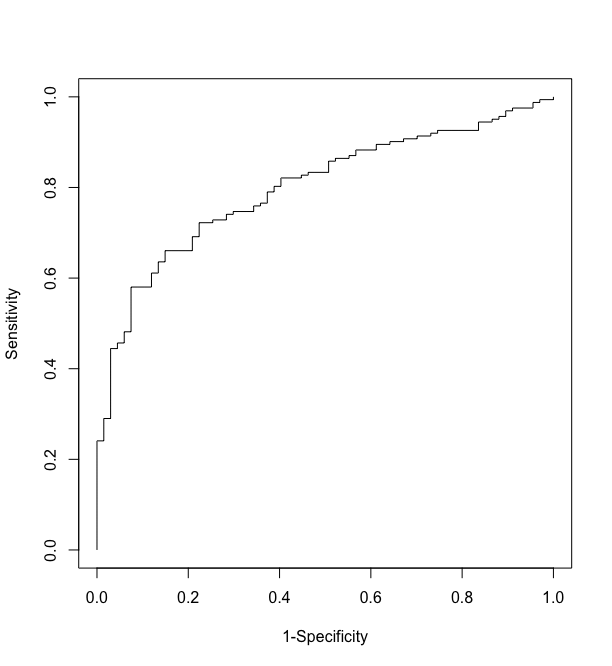


Supplementary Figure S11. ROC curve of the combination of LRG and FC in diagnosing complete ulcer healing in SB and colon patients.

AUC: 0.80

Point estimate of AUC and the 95% confidence interval obtained via cross validation and the boot-strap method: 0.79 (95%CI: 0.74–0.86).

Section S6. Results of additional analysis #4

Results of additional analysis #4: diagnosing endoscopic remission in SB patients


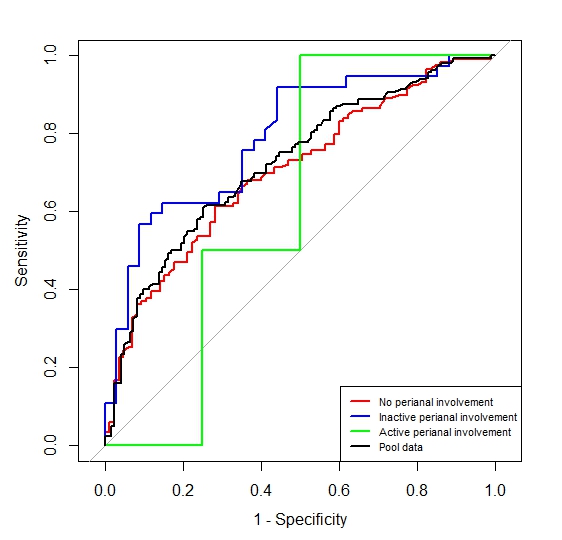


Supplementary Figure S12. ROC curves with stratification of perianal involvement in diagnosing endoscopic remission in SB patients.

AUC

No perianal involvement: 0.70 (95%CI: 0.63–0.77)

Inactive perianal involvement: 0.79 (95%CI: 0.68–0.90)

Active perianal involvement: 0.63 (95%CI: 0.14–1.00)

Pool data: 0.72 (95%CI: 0.67–0.78)

Results of additional analysis #4: diagnosing complete ulcer healing in SB patients


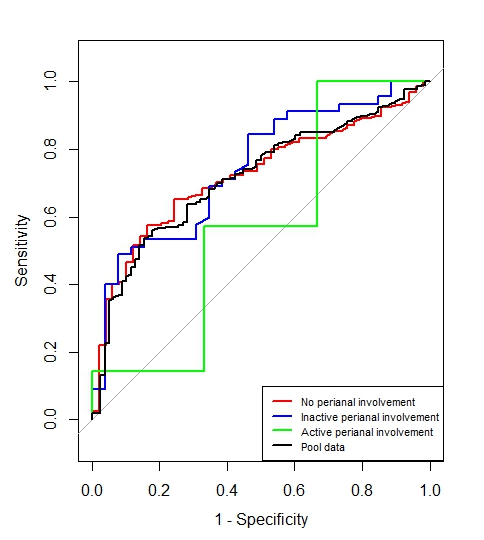


Supplementary Figure S13. ROC curves with stratification of perianal involvement in diagnosing complete ulcer healing in SB patients.

AUC

No perianal involvement: 0.72 (95%CI: 0.65–0.80)

Inactive perianal involvement: 0.74 (95%CI: 0.63–0.86)

Active perianal involvement: 0.57 (95%CI: 0.05–1.00)

Pool data: 0.72 (0.65–0.78)

Results of additional analysis #4: diagnosing endoscopic remission in SB and colon patients


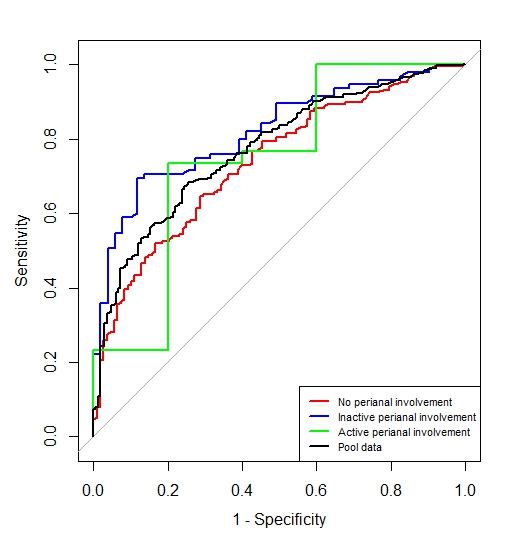


Supplementary Figure S14. ROC curves with stratification of perianal involvement in diagnosing endoscopic remission in SB and colon patients.

AUC

No perianal involvement: 0.74 (95%CI: 0.68–0.79)

Inactive perianal involvement: 0.82 (95%CI: 0.75–0.89)

Active perianal involvement: 0.75 (95%CI: 0.46–1.00)

Pool data: 0.77 (95%CI: 0.73–0.82)

Results of additional analysis #4: diagnosing complete ulcer healing in SB and colon patients


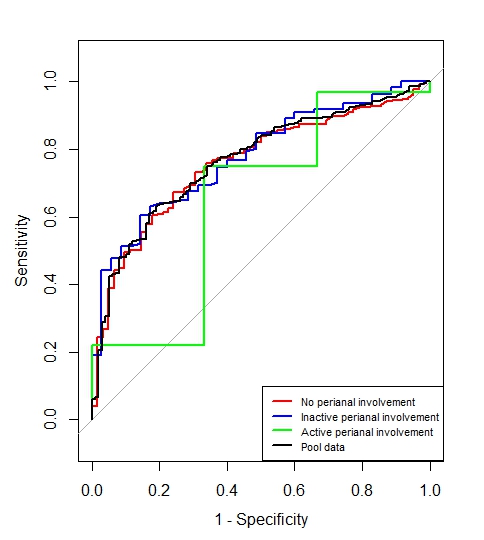


Supplementary Figure S15. ROC curves with stratification of perianal involvement in diagnosing complete ulcer healing in SB and colon patients.

AUC

No perianal involvement: 0.76 (95%CI: 0.70–0.82)

Inactive perianal involvement: 0.78 (95%CI: 0.70–0.86)

Active perianal involvement: 0.65 (95%CI: 0.20–1.00)

Pool data: 0.77 (0.72–0.81)

Section S7. Analyses for the reason why the difference in LRG cut-off values was only 0.4 in SB patients between the definitions of complete ulcer healing and endoscopic remission as endoscopic inactivity.

To provide clarity, we first analyzed the distributions of LRG values in active and inactive disease populations when complete ulcer healing (mSES-CD ≤ 1) was defined as endoscopic inactivity (see Supplementary Figure S16).


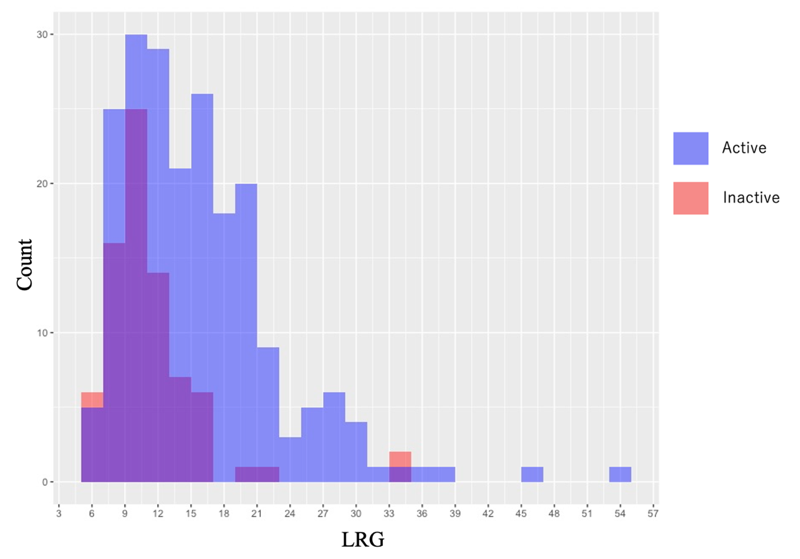


Supplementary Figure S16**.** Distributions of LRG in active and inactive disease populations when complete ulcer healing (mSES-CD ≤ 1) was defined as endoscopic inactivity.

As illustrated in Supplementary Figure S16, the LRG distribution for the inactive population overlaps significantly with that of the active population.

Subsequently, we examined the distributions of LRG values when endoscopic remission (mSES-CD ≤ 3) was defined as endoscopic inactivity (Supplementary Figure S17).


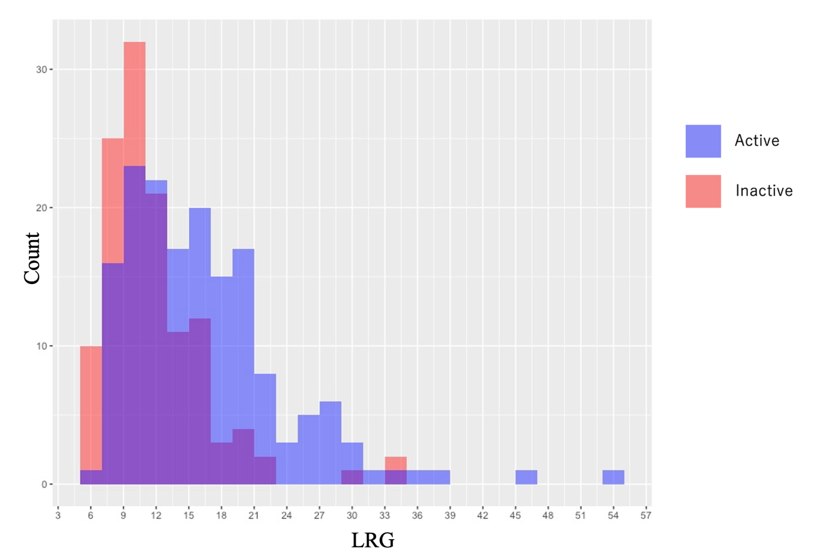


Supplementary Figure S17**.** Distributions of LRG in active and inactive disease populations when endoscopic remission (mSES-CD ≤ 3) was defined as endoscopic inactivity

Supplementary Figure S17 shows a more pronounced peak in the inactive disease population’s LRG distribution, suggesting a favorable trend. In the case of complete ulcer healing (mSES-CD ≤ 1), patients with mSES-CD scores of 2 and 3 were classified under the active disease population. Conversely, when endoscopic remission (mSES-CD ≤ 3) was defined as inactivity, these patients were included in the inactive disease population. The cutoff values were determined to achieved a sensitivity of 80%, reflecting the percentage of biomarker-positive results in the active disease population. As this calculation does not involve the inactive disease population, the focus here is only on the LRG distributions in the active disease population.

When patients with mSES-CD scores of 2 and 3 under the complete ulcer healing (mSES-CD ≤ 1) criterion were reclassified as inactive population, the LRG distribution in the active disease population was consistent with the distribution observed in the active disease population under the endoscopic remission (mSES-CD ≤ 3) criterion.

To enhance clarity, Supplementary Figure S18 exclusively depicts the LRG distribution in the active disease population under the complete ulcer healing (mSES-CD≤1) criterion.


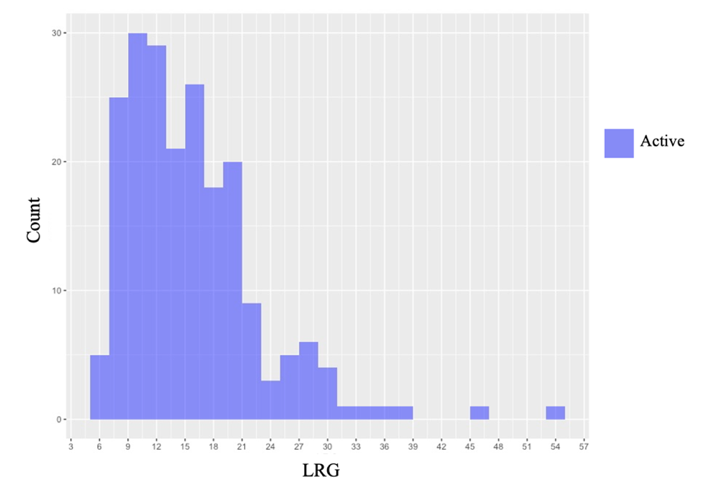


Supplementary Figure S18**.** LRG distribution in the active disease population under the complete ulcer healing (mSES-CD ≤ 1) criterion

The distributions of LRG among patients with mSES-CD scores of 2 and 3 were analyzed to identify differences in cutoff values under the two endoscopic inactivity criteria. The cutoff value of LRG was defined as the 80th percentile of the active population’s LRG distribution. The ideal LRG distribution for patients with mSES-CD scores of 2 and 3, which maximized the difference between the cutoff values, is shown in Supplementary Figure S19.


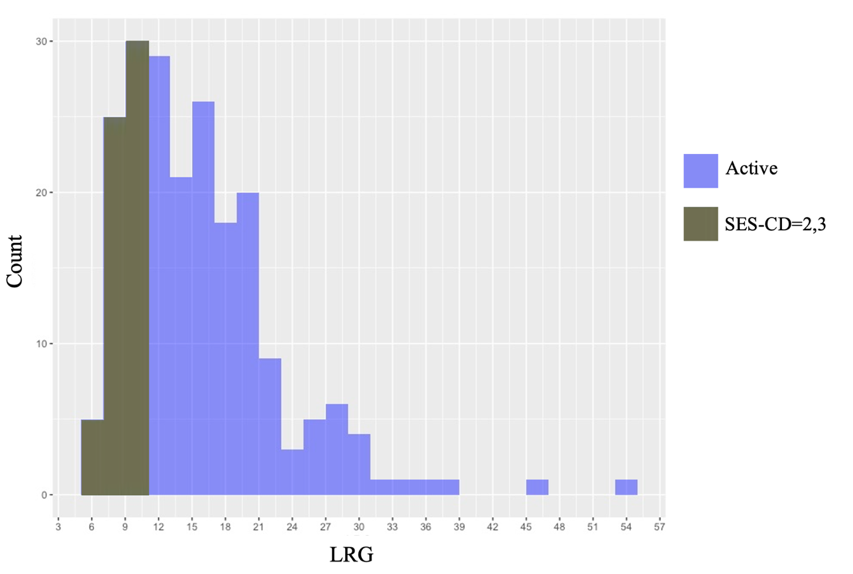


Supplementary Figure S19. Ideal distribution of LRG in patients with mSES-CD scores of 2 and 3, where the difference between the cutoff values of LRG for complete ulcer healing (mSES-CD ≤ 1), defined as endoscopic inactivity, and endoscopic remission (mSES-CD ≤ 3), also defined as endoscopic inactivity, is widest

If the LRG cutoff values for endoscopic remission and complete ulcer healing differ slightly, two possibilities arise: (1) the distribution of LRG in patients with mSES-CD scores of 2 and 3 deviates significantly from the ideal LRG distribution and (2) the number of patients with mSES-CD scores of 2 and 3 is insufficient to generate a meaningful impact on the cutoff values.

The actual LRG distributions in patients with mSES-CD scores of 2 and 3 in Supplementary Figure S20.


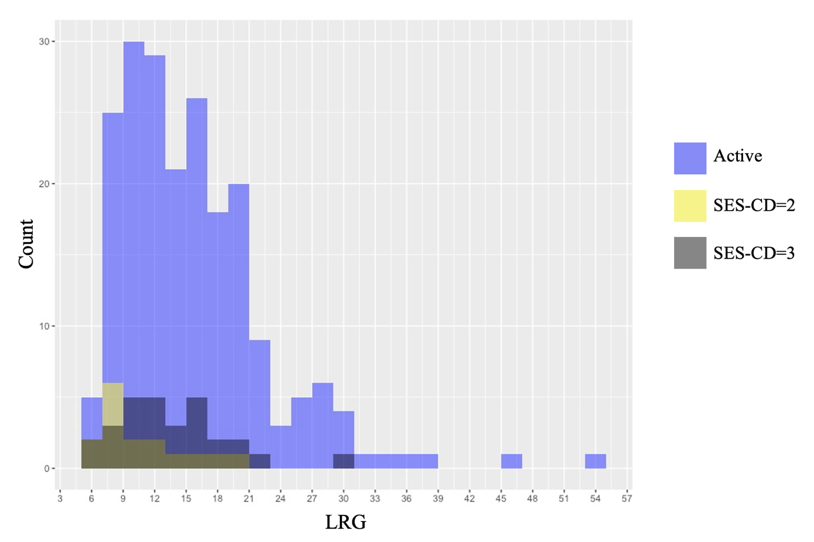


Supplementary Figure S20.Actual LRG distributions in patients with mSES-CD scores of 2 and 3

As depicted in Supplementary Figure S20, the LRG distribution in patients with an mSES-CD score of 2 closely approximates the ideal distribution, though it is right-skewed. In contrast, the distribution in patients with an mSES-CD score of 3 deviates significantly from the ideal. Furthermore, the sample sizes of patients with mSES-CD scores of 2 and 3 are insufficient to exert a significant influence on the LRG cutoff values.

Section S8. Comparison of our study and previous LRG studies.

Supplementary Table S1. Comparison of our study and the previous LRG studies on cut-off values and diagnostic abilities.

|  | The definition of endoscopic inactivity | LRG cut-off value (μg/ml) | Sensitivity/  Specificity | AUC |
| --- | --- | --- | --- | --- |
| Kawamoto2024 | mSES-CD≦3 | 13 | 0.78/0.80 | 0.84 |
| Matsumoto 2023 | SES-CD=0 | 14.3 | 0.89/0.63 | 0.80 |
| Kawamura 2022 | aSES-CD≦2* | 8.9 | 0.93/0.83 | 0.90 |
| Kawamoto 2022 | mSES-CD≦3 | 13.4 | 0.79/0.82 | 0.85 |
| Yoshida 2022 | SES-CD≦2 | 12.6 | 0.78/0.90 | 0.89 |
| Abe 2022 | SES-CD≦2 | 13.6 | 0.84/0.85 | 0.89 |
| Shimoyama 2022 | SES-CD≦2 | 13.3 | 0.87/0.62 | 0.79 |
| Yasutomi 2021 | mSES-CD=0 | 13 | 0.84/0.73 | 0.82 |
| Our study  (SB patients) | mSES-CD≦1 | 10.1 | 0.81/0.46 | 0.72 |
| Our study  (SB patients) | mSES-CD≦3 | 10.5 | 0.80/0.47 | 0.72 |
| Our study  (SB and colon patients) | mSES-CD≦1 | 10.6 | 0.81/0.54 | 0.77 |
| Our study  (SB and colon patients) | mSES-CD≦3 | 11.1 | 0.82/0.55 | 0.77 |

*a-SES-CD: Applied SES-CD

Supplementary Table S2. Inclusion and exclusion criterion of the previous LRG studies and our study.

|  | Inclusion/Exclusion criterion |
| --- | --- |
| Kawamoto 2024 | ・Serum LRG was measured within 8weeks of performing BAE.  ・They excluded patients with colostomy/ileostomy, those with abdominal abscesses, those who were intolerant of or contraindicated to BAE, those on nonsteroidal anti-inflammatories and those with confirmed intestinal infections or malignancies. |
| Matsumoto 2023 | ・They included 141 patients who underwent ileocolonoscopy or transanal enteroscopy within 2 months before and after LRG measurement and had no changes in disease condition or treatment during the same period.  ・Patients who had an evident infection, extraintestinal complications other than mild ones, or anal fistula lesions at the time of blood sampling for LRG measurement and patients who received a coronavirus disease 2019 vaccine in the previous 1 week were excluded. |
| Kawamura 2022 | They included CD patients who underwent endoscopy within 14 days before and after LRG testing at Nagoya university hospital between July 2020 and March 2021. |
| Kawamoto 2022 | ・Serum LRG was measured within 8weeks of performing BAE.  ・They included CD patients with known or suspected small bowel lesions.  ・They excluded patients with purely colonic CD, patients with colostomy/ileostomy, those with abdominal abscesses, those who were intolerant of or contraindicated to BAE, patients in whom the small bowel could not be intubated because of a stricture, and those with confirmed infections or malignancies. |
| Yoshida 2022 | ・Patients with IBD in whom serum LRG and CRP levels were assessed were enrolled.  ・Patients with colon cancer or confirmed inflammation other than IBD were excluded. |
| Abe 2022 | ・Consecutive patients with CD who were initiated onto biologics or switched to other biologics were enrolled and monitored for 52 weeks or until treatment discontinuation.  ・Patients with contraindications to biologic agents, such as those with history of severe infections, were excluded. |
| Shimoyama 2022 | Inclusion criteria:  ・patients with endoscopic and histologic diagnosis of UC or CD.  ・patients who agreed to undergo colonoscopy at entry.  ・patients who agreed to provide stool samples for the measurement of fecal calprotectin.  Exclusion criteria:  ・patients with severe perianal disease  ・patients with jejunostomy, ileostomy, colostomy  ・patients who received nonsteroidal anti-inflammatory drugs at entry. |
| Yasutomi 2021 | ・All IBD patients who underwent colonoscopy or ballon-assisted enteroscopy (BAE) with serum and stool samples obtained on the day endoscopy were considered eligible.  ・The exclusion criteria were insufficient stool collection, having had a colostomy or ileostomy and failure to achieve full endoscopic observation for the patient’s lesions.  ・Patients with other diseases that could affect the levels of LRG and CRP, including extraintestinal complications, collagen disease, heart failure, primary biliary cholangitis, infectious disease, and malignancy, at the time of endoscopy were excluded. |
| Our study | The inclusion criteria:  1) diagnosed with CD based on clinical, endoscopic, and histological criteria  2) with a history of outpatient visits or hospitalization at our hospital and collaborating institutions  3) age≧18 years  4) had LRG values measured by Nanopia (Sekisui Medical, Tokyo, Japan)  5) the ileum can be adequately evaluated via retrograde BAE or long-narrow colonoscopy including selective contrast examination at the deepest point reachable by the endoscope.  The exclusion criteria:  1) having an interval of > 30 days between endoscopy and LRG measurement  2) treatment modification between endoscopy and LRG measurement  3) contraindicated for BAE because of severe SB strictures detected radiographically  4) severe extraintestinal manifestations and anal lesions  5) any cancer. |

References

[1] Camilli C, Hoeh AE, De Rossi G, Moss SE, Greenwood J. LRG1: an emerging player in disease pathogenesis. J Biomed Sci. 2022 Jan 21;29(1):6.
